# Microarray-guided evaluation of the frequency, B-cell origins, and selectivity of human glycan-binding antibodies reveals new insights and novel antibodies

**DOI:** 10.1016/j.jbc.2022.102468

**Published:** 2022-09-08

**Authors:** J. Sebastian Temme, Jennifer A. Crainic, Laura M. Walker, Weizhun Yang, Zibin Tan, Xuefei Huang, Jeffrey C. Gildersleeve

**Affiliations:** 1 Chemical Biology Laboratory, Center for Cancer Research, National Cancer Institute, Frederick, Maryland, USA; 2 Adimab LLC, Lebanon, New Hampshire, USA; 3 Adagio Therapeutics, Inc, Waltham, Massachusetts, USA; 4 Department of Chemistry, Michigan State University, East Lansing, Michigan, USA; 5 Institute for Quantitative Health Science and Engineering, Michigan State University, East Lansing, Michigan, USA; 6 Department of Biomedical Engineering, Michigan State University, East Lansing, Michigan, USA

**Keywords:** antibody, antigen, carbohydrate, carbohydrate-binding protein, glycobiology, microarray, monoclonal antibody, ATCC, American Type Culture Collection, BHI, brain heart infusion broth, CDRH3, complementary determining region 3 of the heavy chain, CETE, carboxyethylthioethyl, DNP-BSA, dinitrophenylated bovine serum albumin, dPNAG, deacetylated PNAG, HC, heavy chain, HIC, hydrophobic interaction chromatography, HRP, horseradish peroxidase, IgG, immunoglobulin G, IgM, immunoglobulin M, KDO, ketodeoxyoctonic acid, 3-deoxy-d-manno-oct-2-ulosonic acid, LC, light chain, LLPC, long-lived plasma cell, mAb, monoclonal antibody, MW, molecular weight, PBST, PBS with Tween-20, PNAG, poly-β-1,6-*N*-acetylglucosamine, PSR, polyselectivity reagent, RT, room temperature, SEC, size-exclusion chromatography

## Abstract

The immune system produces a diverse collection of antiglycan antibodies that are critical for host defense. At present, however, we know very little about the binding properties, origins, and sequences of these antibodies because of a lack of access to a variety of defined individual antibodies. To address this challenge, we used a glycan microarray with over 800 different components to screen a panel of 516 human monoclonal antibodies that had been randomly cloned from different B-cell subsets originating from healthy human subjects. We obtained 26 antiglycan antibodies, most of which bound microbial carbohydrates. The majority of the antiglycan antibodies identified in the screen displayed selective binding for specific glycan motifs on our array and lacked polyreactivity. We found that antiglycan antibodies were about twice as likely than expected to originate from IgG^+^ memory B cells, whereas none were isolated from naïve, early emigrant, or immature B cells. Therefore, our results indicate that certain B-cell subsets in our panel are enriched in antiglycan antibodies, and IgG^+^ memory B cells may be a promising source of such antibodies. Furthermore, some of the newly identified antibodies bound glycans for which there are no reported monoclonal antibodies available, and these may be useful as research tools, diagnostics, or therapeutic agents. Overall, the results provide insight into the types and properties of antiglycan antibodies produced by the human immune system and a framework for the identification of novel antiglycan antibodies in the future.

The immune system produces a diverse assortment of carbohydrate-binding antibodies that are vital for human health (1, 2). For example, antibodies that bind bacterial, fungal, and other microbial polysaccharides provide protection from infections (3). These antibodies can be acquired through natural processes or can be induced through vaccination (4, 5). In fact, Food and Drug Administration–licensed carbohydrate-based vaccines targeting *Streptococcus pneumoniae*, *Haemophilus influenzae* type b, and *Neisseria meningitidis* are administered routinely and have had a major impact on human health (6, 7, 8). Carbohydrate-binding antibodies also influence medical care in other ways (2). For instance, endogenous antibodies to ABO blood group antigens play a critical role in matching donors and recipients for blood transfusions (9). In addition to beneficial effects, antibodies that recognize self-glycans can contribute to autoimmune diseases, such as Guillain–Barre syndrome (10, 11). For these reasons, identifying and studying antibodies that target carbohydrates or glycans is crucial for a complete understanding of the immune system.

While critically important, we know very little about individual antiglycan antibodies and their properties. From profiling serum antibodies on glycan microarrays, we know that many different glycans are recognized by endogenous antibodies (12, 13, 14, 15). However, few details are available for the specific antibodies that bind to those glycans. For example, recognition of the various glycans could be due to: (1) a small number of polyreactive antibodies, (2) a mixture of many different highly selective antiglycan antibodies, or (3) some combination of various polyreactive and selective antibodies. In addition, it is not known if they are highly polyclonal or largely oligoclonal at the sequence level. We know very little about which V, D, and J genes are used to construct antiglycan antibodies, or how the immune system evolves and mutates those antibodies during the course of infection (16). Antiglycan antibodies are often thought to have broad specificity and low affinity, but these generalizations are based on very limited information (17, 18, 19).

A key barrier to answering these questions is a lack of access to individual antiglycan antibodies, especially human antiglycan antibodies. Unlike antibodies to protein or peptide targets, generating monoclonal antibodies (mAbs) to many glycan targets remains an elusive challenge in the field (20). The dearth of antiglycan antibodies can be attributed to a combination of poor immunogenicity and the natural expression of glycans in healthy mammalian tissues, reducing the effectiveness of traditional hybridoma technologies. Alternative routes, such as *in vitro* selection platforms, have had some success in expanding the glycobiology toolbox with synthetic antibodies. Recent examples include the development of smart antiglycan reagents from lamprey lymphocyte receptors, nanobodies from alpacas, and phage display–derived antiheparan sulfate single-chain variable antibody fragment and anti-Tn single-chain variable antibody fragment (21, 22, 23, 24). While these are promising tools, the complexities of glycan structure, inherently weak protein–glycan monovalent interactions, and limited availability to high-purity glycans for selection procedures present unmet challenges in the field. To complicate matters, many of the commercially available antiglycan mAbs are proprietary clones or hybridoma supernatants, lacking both sequence information and affinity and selectivity characterization. As a result, there are only a few hundred human antiglycan antibodies that have been reported to date, and sequence information is only available for about 240 of them (25). Moreover, 70% of the sequenced antibodies target *S. pneumoniae* polysaccharides or HIV glycans, illustrating the limited spectrum of known human antiglycan antibodies. While there are a higher number of reported antiglycan antibodies of mouse origin (including about 350 for which sequences are available), these also target a limited assortment of glycan antigens. Furthermore, using the published antiglycan mouse antibody sequences, germline usage, mutation rates, and even productive glycan epitopes to infer key characteristics of human antiglycan antibodies is of limited benefit to vaccine or therapeutic discovery efforts. Finally, many of the published mAbs are not a random sampling of antibodies produced in a mammal; they are typically isolated following a specific antigen challenge and selected from a broader pool of antibodies based on developability characteristics or clone stability. Therefore, they are not an accurate representation of the repertoire of antiglycan antibodies produced by a mammal.

To gain a better understanding of the types and characteristics of antiglycan antibodies produced in humans, we sought to obtain a sampling of antibodies targeting a variety of glycan antigens. Most screening strategies or mAb development methods are designed to obtain selective and high-quality antibodies to a one or a few antigens (26). For example, one strategy involves using fluorescence-activated cell sorting to isolate B cells that bind a fluorophore-labeled antigen (27, 28). While this strategy works nicely for a small number of antigens, it is impractical for screening against hundreds of antigens and remains particularly difficult for many types of carbohydrates. To overcome these limitations, we screened a panel of antibodies from healthy human subjects against our 800+ component glycan microarray (29, 30).

In a previous study, we cloned and expressed 400 antigen agnostic mAbs from a variety of human B-cell subsets to characterize their biophysical properties (31). Here, we screened 373 of these and an additional 143 antibodies on our glycan microarray to identify a variety of human antiglycan antibodies (Fig. 1). By profiling this collection of antibodies against our library of carbohydrates, we analyzed approximately 400,000 potential antibody–carbohydrate interactions. Since the antibodies were isolated from different subsets of B cells, the study also allowed us to evaluate which types of B cells most frequently produce antiglycan antibodies. The results provide insight into the cellular origins and binding specificities of human antiglycan antibodies and have implications for developing antiglycan antibody diagnostics and therapeutics.

**Figure 1:**
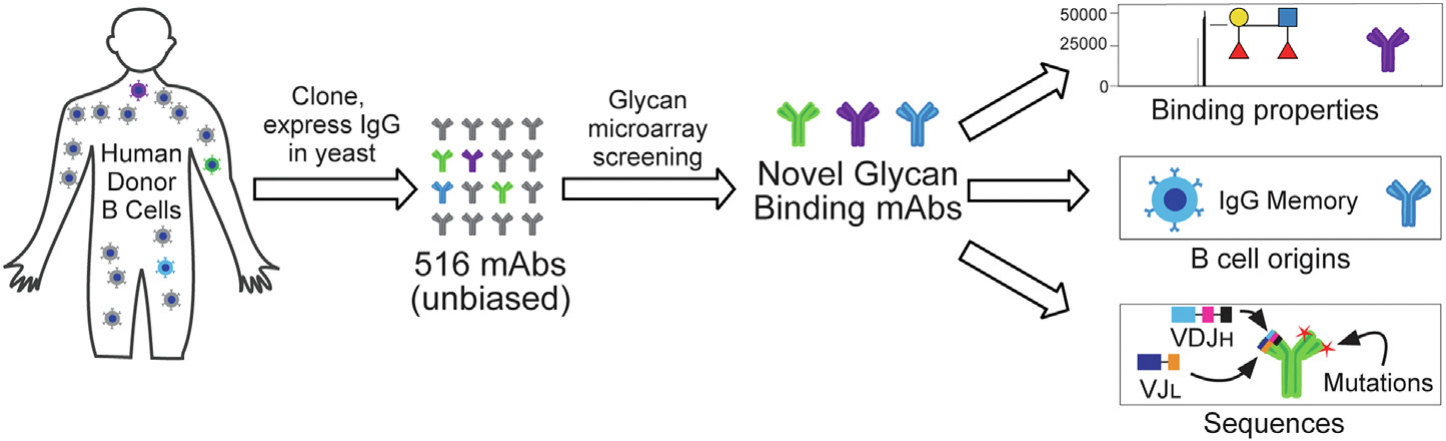
**Flow diagram for screening and analysis**.

## Results

### Characteristics of the antibody panel

A panel of human mAbs composed of 373 previously documented sequences and 143 newly described sequences (see Supporting Excel document) was used for the study (31). mAbs were isolated from long-lived plasma cells (LLPCs; CD19^−^CD38^+^CD138^+^), immunoglobulin M (IgM) memory (IgM^+^CD27^+^), immunoglobulin G (IgG) memory (IgG^+^CD27^+^), and naïve (IgM^+^CD27^−^CD10^−^) B cells using an antigen-agnostic sorting strategy. Natively paired antibody heavy- and light-chain variable regions were cloned and expressed in a proprietary *Streptococcus cerevisiae* strain engineered to express human IgG1 (32). The panel was composed of 516 unique sequence-verified human IgG1 mAbs cloned from 169 IgG memory, 175 LLPCs, 95 IgM memory, and 77 naïve/early emigrant/immature B cells. The early emigrant and immature B cells were two new subsets cloned included in this study. In addition to sequence information, polyreactivity scores, melting temperatures, and hydrophobic interaction chromatography (HIC) retention times were available for most antibodies.

### Development of a pooled screened strategy to identify human antiglycan antibodies

To reduce the total number of microarrays needed for the evaluation, we explored the possibility of screening pools of antibodies. Two of the main concerns with a pooled screen were the hit rate and the potential complications of polyreactive antibodies. If the hit rate is too high, then deconvolution of the pools becomes quite difficult. Moreover, a high proportion of polyreactive antibodies could make the pooled signals nearly impossible to deconvolute. Therefore, we tested approximately 100 antibodies individually to assess the potential hit rate and frequency of polyreactive antibodies. These antibodies were screened at a concentration of 10 μg/ml on a glycan microarray containing approximately 738 different *N*- and *O*-linked glycans, glycans from glycolipids, glycopeptides, and glycoproteins (29).

One of the first barriers we encountered from this original screen was that many antibodies had substantial signals to several components on the array, including highly negatively charged glycosaminoglycan oligomers (3 of the 31 glycosaminoglycans on the array; components #570, #587, and particularly Hep-Octa-GT24 #738), KDOα2-8KDOα2-4KDOα (component #539), dinitrophenylated bovine serum albumin (DNP-BSA) (component #481), and Galα1–4Gal-carboxyethylthioethyl (CETE) (component #31). Several lines of evidence indicated that this was an artifact, potentially caused by antibody aggregation and/or partial denaturation as a result of the antibody isolation process and storage conditions. First, the magnitude of the signals to the glycosaminoglycan oligomers increased with higher negative charge but did not follow a clear structure–activity relationship based on the carbohydrate sequence, suggesting that the interaction was primarily driven by nonspecific charge–charge interactions. Second, approximately half of the antibodies had signals to KDOα2-8KDOα2-4KDOα (component #539) or Galα1–4Gal (component #31) only when presented on a CETE linker. These antibodies failed to bind to three closely related KDO oligosaccharides or other Galα1–4Gal disaccharides presented with different linkers. Third, several negative control antibodies with known protein epitopes were found to bind to neoglycoproteins and other array components following purification and storage. Finally, many of these signals were eliminated when the antibodies were reanalyzed after preparative size-exclusion chromatography (SEC), supporting the theory that these signals were artifacts because of aggregation and/or partially denatured material. We endeavored to characterize these artifacts and the factors contributing to their emergence.

**Figure 2:**
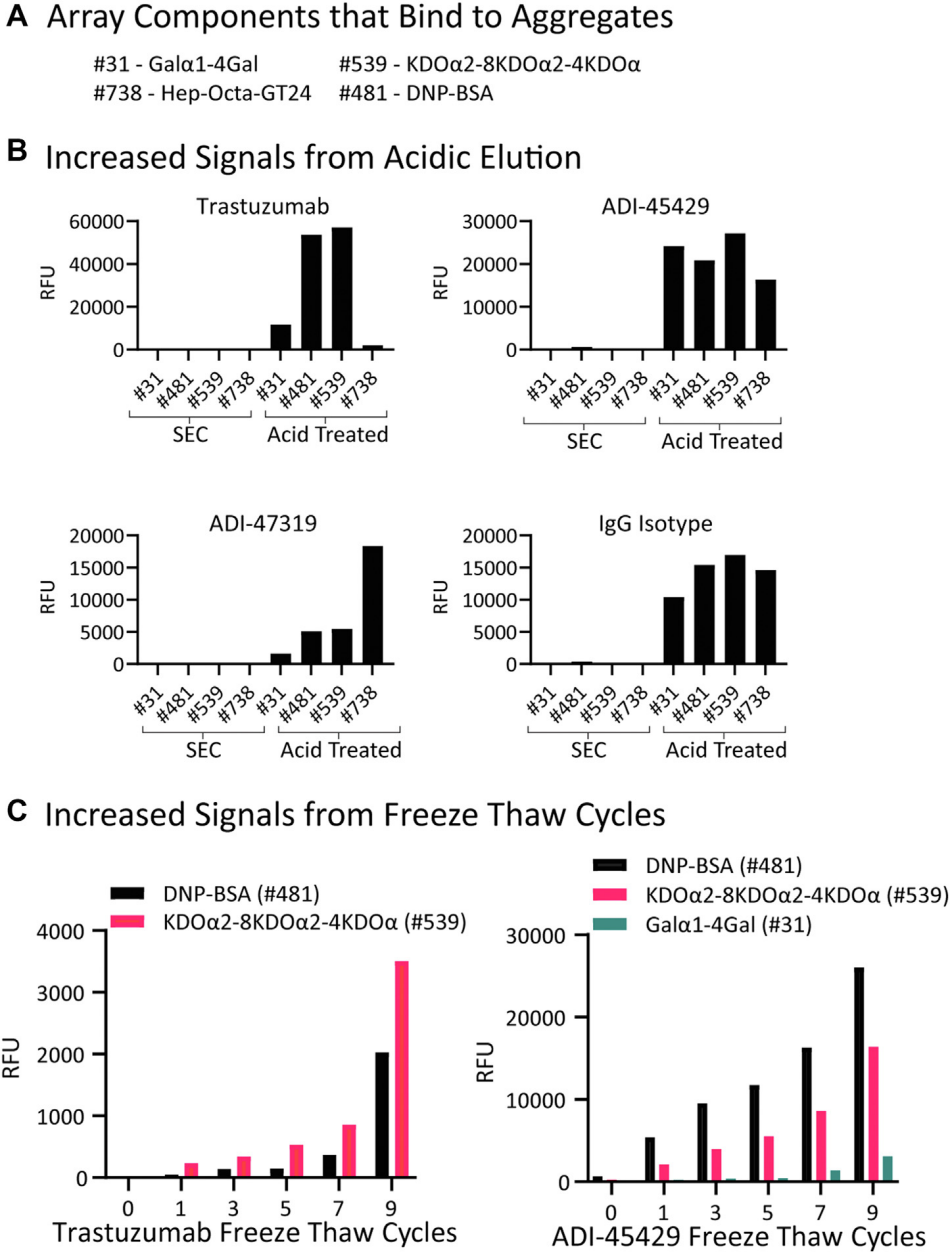
**Selected artifact binding resulting from acidification and freeze–thaw cycling.***A*, legend of array components. *B*, binding events between selected array components and trastuzumab, IgG isotype control, ADI-45429, and ADI-47319 before and after acidification. *C*, additive effect of freeze–thaw cycling results in increased artifact binding with trastuzumab and ADI-45429 as cycle number increases. Signals in relative fluorescence units (RFUs) for antibodies on our 873-component glycan microarray. IgG, immunoglobulin G.

mAb purification *via* capture on Protein A affinity resin and subsequent low pH elution is a common and well-accepted method to obtain research grade mAbs. A number of reports provide evidence that low pH exposure during antibody processing contributes to the appearance of aggregates and misfolded protein (33, 34, 35, 36). To determine if a low pH exposure would produce binding artifacts on the neoglycoprotein microarray, we treated SEC-purified antibodies with an acidic buffer (100 mM glycine, pH 2.5) for 5 min followed by neutralization (1.0 M Tris, pH 8.0). We evaluated the effects of this low pH treatment on trastuzumab, human IgG isotype control, ADI-45429 and ADI-47319. (Note: ADI-45429 was found in the initial screen and thought to bind to many of the polyreactive components [Fig. 2*A*] and ADI-47319 was identified as a glycan binding mAb from the screen). As shown in Figure 2*B*, all SEC-purified mAbs tested initially showed little to no binding to Hep-Octa-GT24 (component #738), KDOα2-8KDOα2-4KDOα (component #539), DNP-BSA (component #481), or Galα1–4Gal-CETE (component #31). Acidification and subsequent neutralization of trastuzumab resulted in significant binding to array components #539 and #481. Elevated levels to #31 and #738 were also observed. The number of extra signals and their intensity was dependent on the individual antibody tested. IgG isotype control and ADI-45429 developed strong signals to all four array components. Acidification of ADI-47319 showed significant binding to the highly charged GAG #738, while also having moderately increased signals to other components. Analytical SEC traces are shown in Fig. S1. Traces in *red* suggest that higher molecular weight (MW) species are enriched in the acid-treated samples relative to the original preparations.

Antibody storage was another potential source of aggregation or misfolding. To evaluate storage conditions, we diluted SEC-purified mAbs to 1 mg/ml into PBS and subjected them to nine rounds of freeze–thaw cycles. Trastuzumab and ADI-45429 were analyzed on the array following each round of freeze–thaw cycle. As shown in Figure 2*C*, both antibodies developed significant signals to KDOα2-8KDOα2-4KDOα (component #539) and DNP-BSA (component #481). Signal strength increased with each round of freeze–thaw cycle. Images of trastuzumab on the array in Fig. S2 demonstrate the appearance of signals to DNP-BSA and KDOα2-8KDOα2-4KDOα following freeze–thaw cycles (Fig. S2*B*) when compared with the SEC-purified mAb on the array (Fig. S2*A*). HPLC SEC traces of the freeze–thaw cycled mAbs (*green traces*, Fig. S1) were similar to the unfrozen mAbs, indicating that the aggregation/misfolding was nearly undetectable using traditional HPLC analytical SEC methods despite producing significant artifacts on the array. We next investigated storage additives in an attempt to prevent these artifacts from emerging in the future. Freeze–thaw cycling was performed with 1% BSA and 0.2 M trehalose, two commonly used stabilizers. Gratifyingly, both BSA and trehalose offered significant protection from spurious signals on the array following freeze–thaw cycles. Data for #539 and #481 are shown in Fig. S3.

Because these experiments suggest that binding to certain array components indicates low-quality antibody, we opted to exclude antibodies that only bound to several of the most highly charged glycosaminoglycans (3 of the 31 glycosaminoglycans on the array; components #570, 587, and 738), Galα1–4Gal-CETE #31, or KDOα2-8KDOα2-4KDOα #539 (but not the other KDO-containing glycans on the array) when identifying antiglycan antibodies from the panel. It is possible that these exclusion criteria may have precluded the identification of antiglycan antibodies that truly target these glycans, but this was necessary to avoid numerous false positives. We reasoned that any antiglycan antibodies should have produced signals to some of the structurally related components on the array and would have been further characterized. From our initial screen and the polyreactivity scores for the antibodies, we anticipated an antiglycan antibody hit rate of about 1 to 5% and a frequency of polyreactive antibodies of about 1 to 5%. Therefore, we decided to screen groups of antibodies in a two-dimensional 12 × 16 pooled matrix, with each antibody screened at a concentration of approximately 10 μg/ml. In this pooled strategy, antibodies would be screened twice in separate groups of 12 and 16 antibodies. With a combined 2 to 10% hit rate, deconvolution of positive hits present in both pools would be feasible.

### Identification of antiglycan antibodies using the microarray screen

Using the pooled antibody strategy, we screened the full panel of 516 antibodies on our 738-component microarray. From this screen, we identified 24 candidate antiglycan antibodies. During the course of these studies, we obtained a variety of additional glycans and then printed new glycan microarrays with 816 array components. To evaluate reproducibility of the pooled screen and determine if there were any antibodies to the new components, we repeated the screen on the 816-component microarray. Each of the hits from the original screen were identified again, along with two additional antibodies that bound newly acquired glycans that were not present on the original 738-component array. These results demonstrate good reproducibility of the screen and suggest that there may be a significant number of additional antiglycan antibodies in the panel that recognize glycans not present on our microarray.

Each of the 26 candidate hits from the pooled screen was evaluated individually on the array at a concentration of 10 μg/ml. Furthermore, each of the hits was also re-expressed recombinantly in mammalian cells as a human IgG1, purified by protein A, and retested on the array at four concentrations ranging from 1.5 to 100 μg/ml. Overall, we obtained 17 antibodies with an estimated apparent *K*_*D*_ value ≤150 nM to at least one glycan, five antibodies with weak but measurable binding to one or more glycans, two antibodies that were polyreactive, and two antibodies with inconsistent results. The two inconsistent antibodies displayed good binding using the original samples produced in yeast but no binding when they were re-expressed in mammalian cells. Taken together, the screen had an overall hit rate of ∼5% and a hit rate of “good” antiglycan antibodies (*i.e.*, antibodies with apparent *K*_*D*_ values ≤150 nM) of around 3%.

### Human antiglycan antibodies recognize a diverse set of glycans

**Figure 3:**
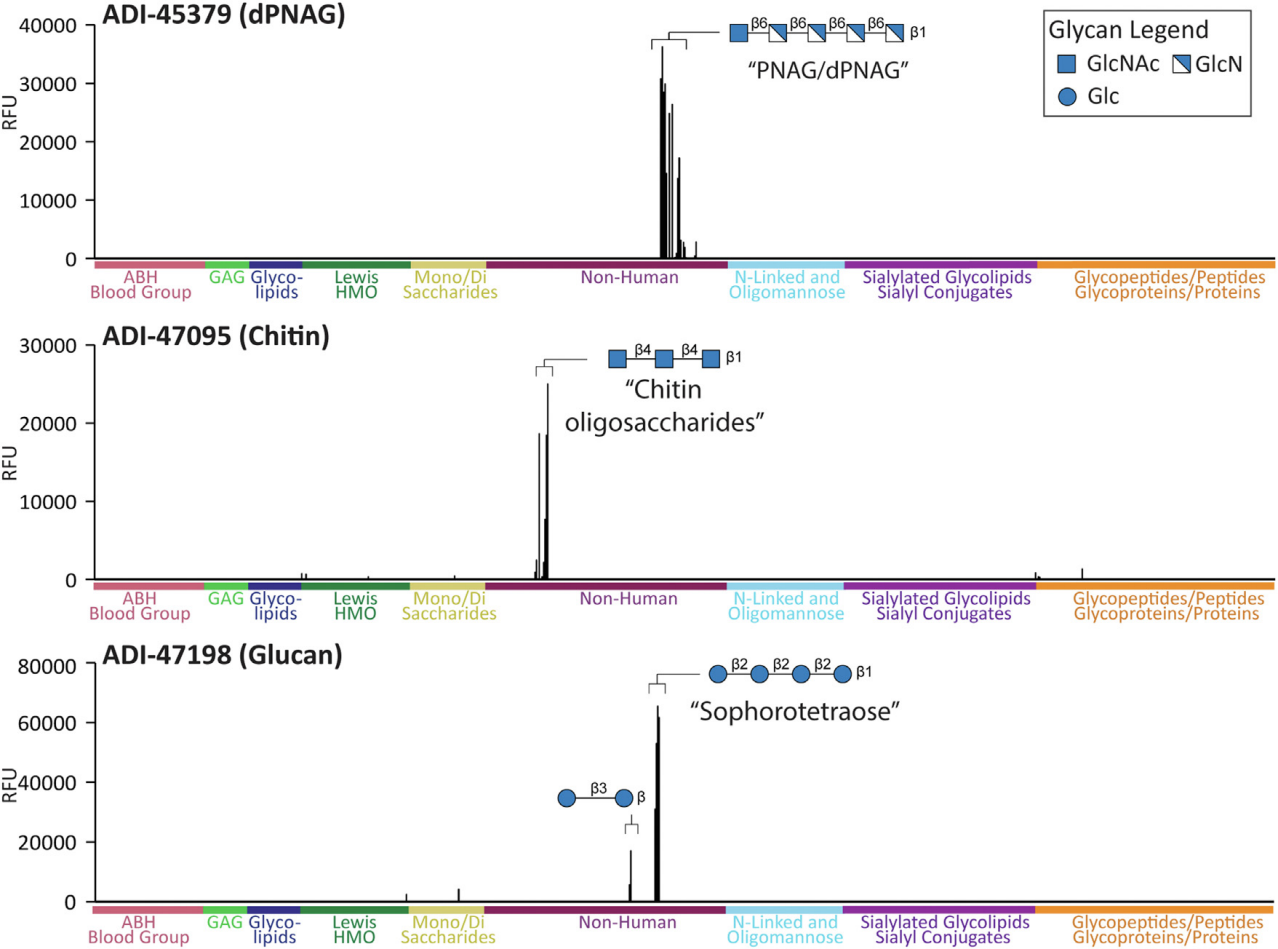
**Binding profiles for ADI-45379, ADI-47095, and ADI-47198.** Signals in relative fluorescence units (RFUs) for antibodies on our 873-component glycan microarray. Data shown at the following concentrations: ADI-45379 at 42 nM, ADI-47095 at 167 nM, and ADI-47198 at 42 nM. Positive and negative controls have been excluded. Glycan symbol structures were created in GlycoGlyph (33).

**Figure 4:**
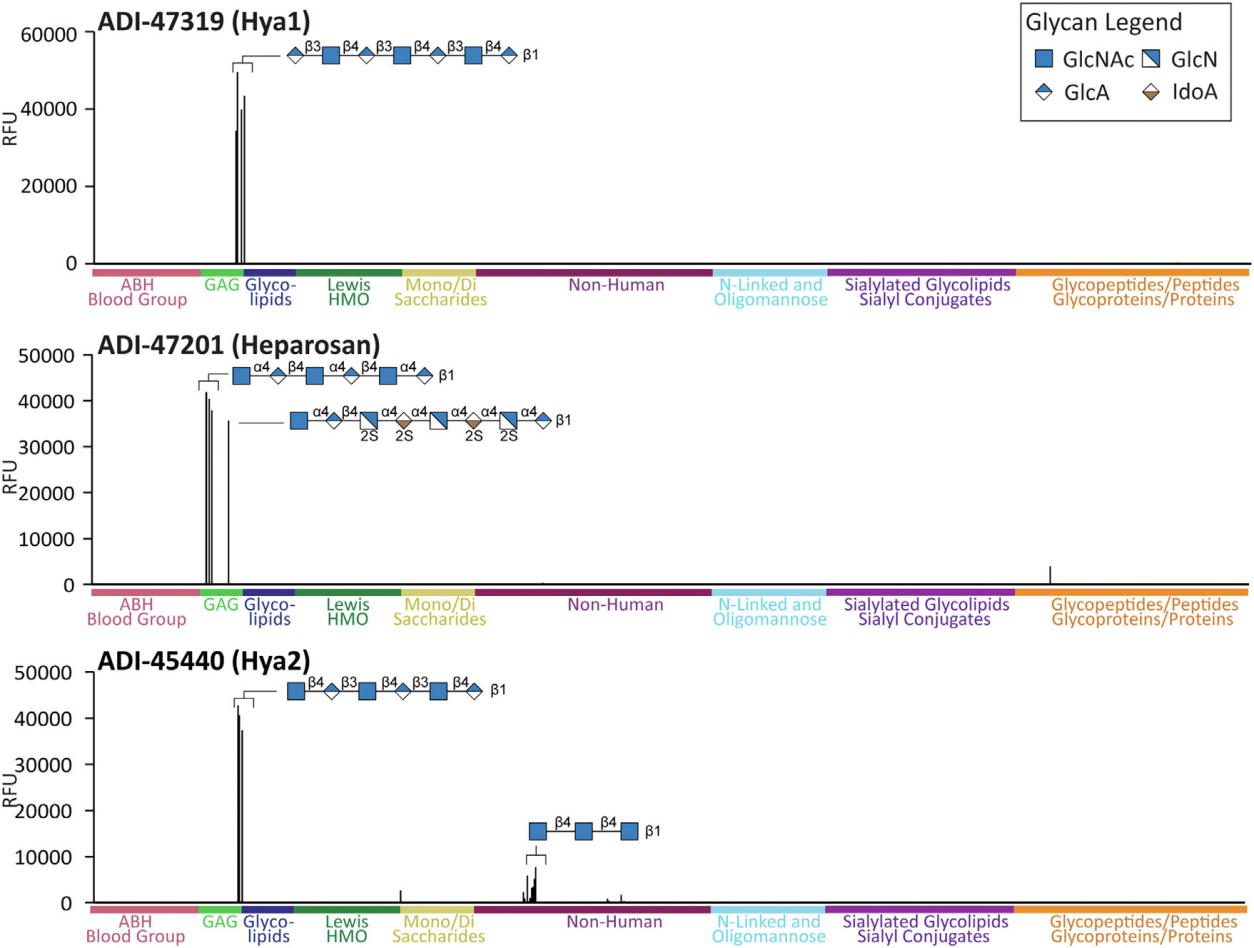
**Binding profiles for ADI-47319, ADI-47201, and ADI-45440.** Signals in relative fluorescence units (RFUs) for antibodies on our 873-component glycan microarray. Data shown at the following concentrations: ADI-47319 at 167 nM, ADI-47201 at 42 nM, and ADI-45440 at 10 nM. Positive and negative controls have been excluded. Glycan symbol structures were created in GlycoGlyph (33).

**Figure 5:**
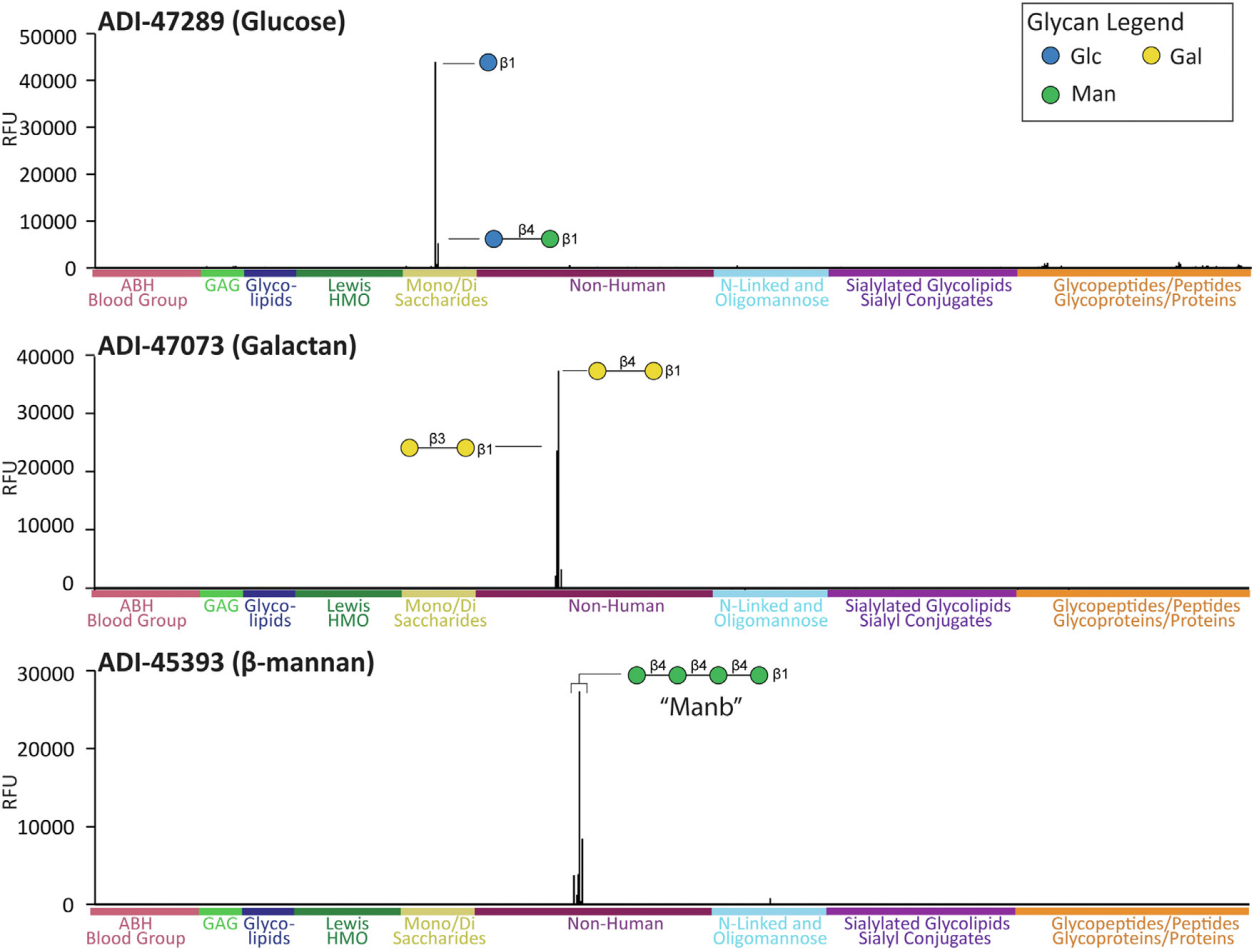
**Binding profiles for ADI-47289, ADI-47073, and ADI-45393.** Signals in relative fluorescence units (RFUs) for antibodies on our 873-component glycan microarray. Data shown at the following concentrations: ADI-47289 at 42 nM, ADI-47073 at 167 nM, and ADI-45393 at 167 nM. Positive and negative controls have been excluded. Glycan symbol structures were created in GlycoGlyph (33).

The antibodies isolated in the screen targeted a wide variety of glycan antigens (Table 1). The majority of antibodies bound glycans found in bacterial and/or fungal cell walls, but several bound mammalian and plant glycans. Later, we describe the targets and selectivities for a subset of the antiglycan antibodies. Bar graphs illustrating binding profiles for the 24 consistent antibodies can be found in Figure 3, Figure 4, Figure 5 and Figs. S4–S6. In cases where a natural source of a glycan was readily available, we also carried out additional binding studies for validation.

**Table 1:** Human antiglycan mAbs identified by pooled matrix neoglycoprotein microarray screen

Name	B-cell subset	Glycan name	Glycan determinant	App *K*_*D*_ (nM)	Reactivity score	HIC retention
Moderate- to high-affinity glycan-binding mAbs						
ADI-45474	LLPC	Gb4 and iso-Gb4	GalNAcβ1–3Galα1–4Galβ1–4GlcNAcβ	∼40	0.03	ND
ADI-47319	IgG memory	Hyaluronic acid	Terminal GlcAβ1–3GlcNAcβ1–4GlcAβ1–3	∼20	0.00	ND
ADI-47213	IgM memory	Blood group B	Galα1–3(Fucα1–2)GlcNAc	∼150	0.00	9.08
ADI-47201	IgG memory	Heparosan	GlcNAcα1–4GlcAβ1–4	∼20	0.00	8.94
ADI-47289	IgG memory	Glucose	Glc-β	∼5	0.00	8.88
ADI-47217	IgG memory	Hyaluronic acid	Terminal GlcAβ1–3GlcNAcβ1–4GlcAβ1–3	∼180	0.00	9.10
ADI-47073	IgG memory	Galactan	Galβ1–4Galβ	∼40	0.00	8.83
ADI-45440	IgG memory	Hyaluronic acid	Terminal GlcNAcβ1–4GlcAβ1–3GlcNAcβ	<5	0.00	9.02
ADI-47180	IgG memory	Lewis B, CF3-LeY	Fucα1–2Galβ1–4(Fucα1–3)GlcNAc	∼100	0.00	8.73
ADI-47173	IgG memory	Xylan/cellulose	Either Glcβ1–4Glcβ or Xylβ1–4Xylβ	∼40	0.00	8.47
ADI-47119	IgG memory	LNT/Lewis C/type 1	Terminal Galβ1–3GlcNAcβ	∼50	0.00	9.26
ADI-47133	LLPC	GlcNAcα1–4Gal	GlcNAcα1–4Gal	∼150	0.00	9.57
ADI-47114	LLPC	Chitin	GlcNAcβ1–4GlcNAcβ	∼80	0.00	9.24
ADI-45379	IgG memory	PNAG/dPNAG	GlcNβ1–6GlcNβ	∼10	0.00	8.79
ADI-45393	IgM memory	Linear β-mannan	Manβ1–4Manβ	∼150	0.00	8.84
ADI-47198	IgG memory	β1–2 glucan	Glcβ1–2Glcβ	<5	0.00	8.93
ADI-47095	LLPC	Chitin	GlcNAcβ1–4GlcNAcβ	∼80	0.09	9.02
Weak glycan-binding mAbs						
ADI-45404	LLPC	GA2di	GalNAcβ1–4Galβ	>1000	0.00	8.86
ADI-45497	IgM memory	Sialyl-LacNAc	Neu5Acα2-6[Galβ1–4GlcNAcβ1–3)2β	>1000	0.39	12.74
ADI-47063	IgG memory	Lactose/Lewis A	Galβ1–4Glcβ	>1000	0.00	8.76
ADI-47227	LLPC	SLeC/GD3(9-OAc)	Neu5Acα2-3Galβ1–3GlcNAcβ	>1000	0.04	9.25
ADI-47299	LLPC	MUC4 glycopeptide	TSSA-(Galβ1–3GalNAcα1)S-TGHATPLPVTD	>1000	0.13	9.05
Polyreactive mAbs						
ADI-46714	IgM memory	Multiple	Multiple	ND	0.11	ND
ADI-45499	IgM memory	Multiple and DNP	Multiple and DNP	ND	0.90	12.22
Bound when expressed in yeast						
ADI-75423	LLPC	Sialic acid	Neu5Ac	ND	0.38	ND
ADI-45370	IgG memory	Heparosan	GlcNAcα1–4GlcA	ND	0.00	8.85

Abbreviation: ND, not determined.

### Antibodies targeting microbial glycans

#### ADI-45379, a deacetylated poly-β-1,6-*N*-acetylglucosamine-binding antibody

Poly-β-1,6-*N*-acetylglucosamine (PNAG) is a polysaccharide produced by a wide variety of bacteria and fungi that is especially prevalent in bacterial biofilms (37). It is composed of GlcNAcβ1–6GlcNAcβ-repeat units, and approximately 5 to 30% of the GlcNAc residues are deacetylated to produce glucosamine residues (GlcN) within the polymer. Deacetylation is critical for biofilm formation, and deacetylated PNAG (dPNAG) is preferred over PNAG for mAb therapeutics and vaccine development (38, 39). Antibodies to PNAG are abundant in human serum. In addition, an antibody that binds dPNAG (F598) is currently in phase II clinical trials for treating infections in intensive care units, and a vaccine that induces antibodies to PNAG is in phase II clinical trials for *S. pneumoniae* infections (NCT01389700; https://clinicaltrials.gov/ct2/show/NCT01389700?term=SAR279356&rank=1).

Our glycan microarray contained a set of 32 PNAG pentasaccharides, encompassing all possible combinations of acetylation/deacetylation. Antibody ADI-45379 was highly selective for PNAG glycans on our array (Fig. 3). It bound best to highly dPNAG pentasaccharides, such as GlcNβ1–6GlcNβ1–6GlcNβ1–6GlcNβ1–6GlcN, GlcNAcβ1–6GlcNβ1–6GlcNβ1–6GlcNβ1–6GlcN, and GlcNβ1–6GlcNβ1–6GlcNβ1–6GlcNβ1–6GlcNAc. ADI-45379 did not bind fully acetylated PNAG pentasaccharide or pentasaccharides with only one GlcN residue, even at the highest concentration tested (100 μg/ml). ADI-45379 had an estimate apparent *K*_*D*_ value of 10 nM for PNAG pentasaccharides with four or five GlcN residues.

**Figure 6:**
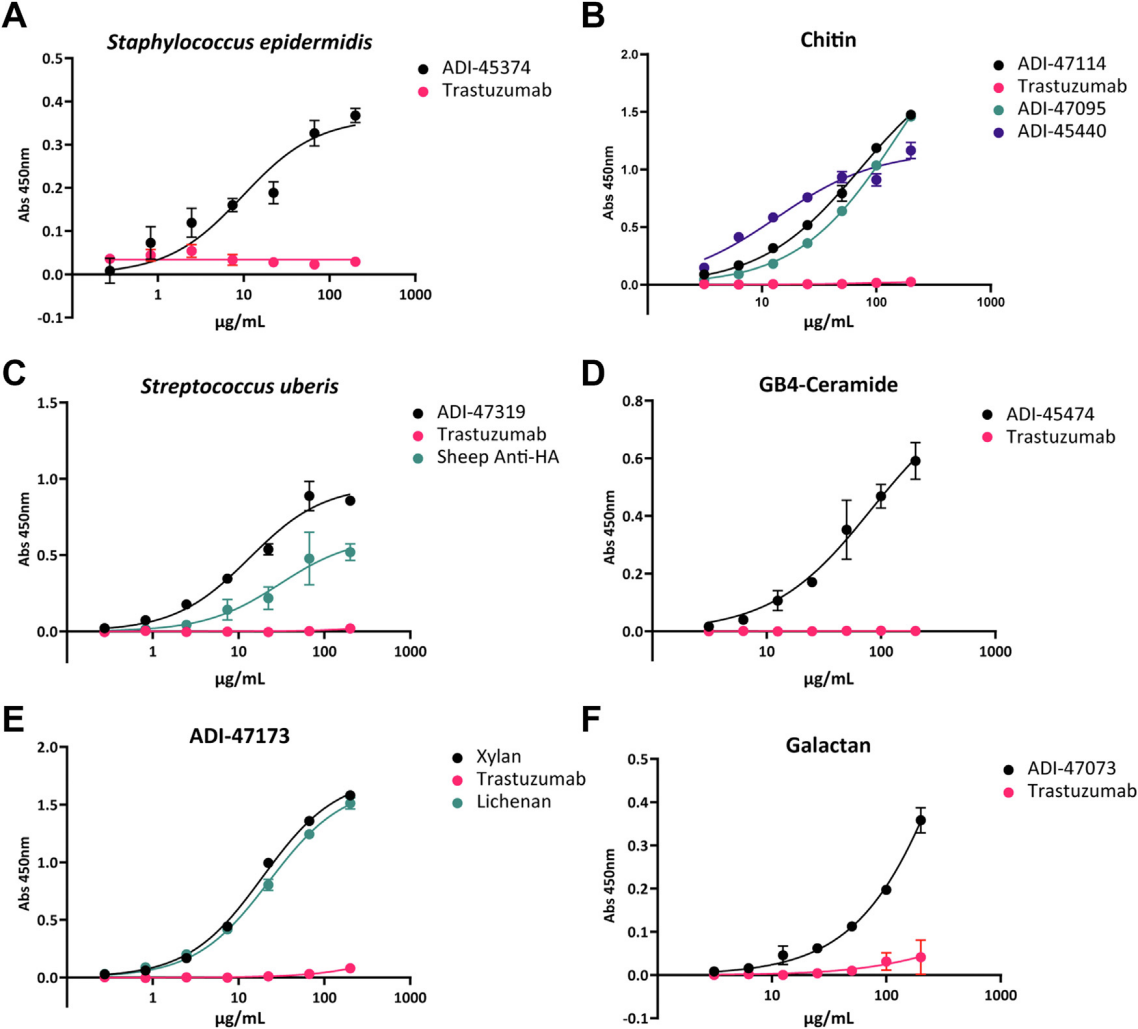
**Validation of monoclonal antibody (mAb) binding to natural glycans and bacterial cultures.** Signals in mean absorbance (Abs) read at 450 nm. Fixed bacteria or glycans plated in 96-well plates. Antibody dilution curves in triplicate shown, trastuzumab used as a negative control. *A*, ADI-45374 anti-PNAG properties evaluated against the PNAG-producing bacteria *Staphylococcus epidermidis*. *B*, ADI-47114, ADI-47095, and ADI-45440 bound chitin from shrimp shells. *C*, ADI-47319 and sheep anti–hyaluronic acid polyclonal antibody evaluated for hyaluronic acid binding against the hyaluronic acid capsule forming bacteria *Streptococcus uberis*. *D*, ADI-45474 binding to Gb4-ceramide. *E*, ADI-47173 binds strongly to both xylan and lichenan when compared with trastuzumab. *F*, ADI-47073 binding to galactan. PNAG, poly-β-1,6-*N*-acetylglucosamine.

For additional validation, we evaluated binding of ADI-45379 to a strain of PNAG producing *Staphylococcus epidermidis* (Winslow and Winslow) Evans (American Type Culture Collection [ATCC]; catalog no.: 35984). Planktonic overnight cultures were washed and subsequently fixed in methanol. The fixed cells were aliquoted to a 96-well plate, dried, and then rehydrated in water. ADI-45379 bound to the plated *S. epidermidis* bacterial cultures as shown in Figure 6*A*.

#### ADI-47114 and ADI-47095, chitin-binding antibodies

Chitin is a polysaccharide composed of repeating GlcNAcβ1–4GlcNAcβ units. It is one of the main constituents of fungal cell walls, making up 2 to 15% of the mass (40). Furthermore, it is a critical component of pathogenic fungi and considered a potential therapeutic target (41). Prior studies of individual human serum samples have shown that antichitin antibodies are among the most abundant and the most commonly observed antiglycan antibodies in human serum (15, 42, 43, 44). In addition, antichitin antibodies have been shown to provide protection from *Aspergillus fumigatus*–induced airway disease in a mouse model (45).

Antibodies ADI-47114 and ADI-47095 display very similar binding properties (Figs. 3 and S5). Both bound a variety of GlcNAcβ1–4 oligomers of varying length. Binding to the chitobiose disaccharide (GlcNAcβ1–4GlcNAcβ) was only about twofold to threefold lower than binding to the trisaccharide, and the binding to the trisaccharide was equivalent to the tetrasaccharide and pentasaccharide. Both antibodies also had some reactivity with glycopeptides with core 4 glycan (GlcNAcβ1–3(GlcNAcβ1–6)GalNAcα1-Ser/Thr) and core 3 glycan (GlcNAcβ1–3GalNAcα1-Ser/Thr). ADI-47095 displayed better selectivity than ADI-47114 for chitin oligosaccharides over core 3/4 glycopeptides. For both antibodies, the estimate apparent *K*_*D*_ value for the best chitin oligomer was about 80 nM.

We next evaluated recognition of chitin polysaccharide in a separate assay. Chitin and chitosan were dried onto ELISA plates, and antibodies ADI-47114 and ADI-47095 were evaluated for binding relative to trastuzumab control. As shown in Figure 6*B*, both antibodies bound to the chitin-coated plates. The antibodies showed good selectivity for chitin, with no detectable binding to the deacetylated chitin analog, chitosan (see Supporting Excel File).

#### ADI-47198 and ADI-47289, glucose/glucan-binding antibodies

Polysaccharides and oligosaccharides containing beta-linked glucose residues are abundant in a wide range of species, including cell walls of yeast/fungi, polysaccharide capsules of bacteria, algae, and various plants (*e.g.*, seaweed, barley) (40). The most abundant glucans are composed of Glcβ1–3Glcβ or Glcβ1–4Glcβ repeating units that may or may not have Glcβ1–6Glc branching. Glucans containing β1–2 linkages are found in cyclic glucans produced by some *Agrebacterium*, *Rhizobium*, and *Brucella* bacteria (46). Glcβ1–2Glc branching can be found in *S. pneumoniae* type 37 polysaccharide, and sophorolipids found in some yeast contain a Glcβ1–2Glcβ disaccharide attached to a lipid (47).

Antibody ADI-47198 displayed high selectivity for β1–2 linked glucans, with some modest reactivity to β1–3 linked glucans (Fig. 3). Binding to the β1–2 linked glucan sophorotetraose was saturated at the lowest concentration tested, indicating that the apparent *K*_*D*_ value is <5 nM. ADI-47198 did not bind β1–4 linked glucans, alpha-linked glucans, and glucose monosaccharides. We are not aware of any mAbs that have similar selectivity as ADI-47198.

Antibody ADI-47289 displayed remarkable selectivity for Glc-β monosaccharide with a long flexible linker (Fig. 5). The apparent *K*_*D*_ value for this sugar was 5 nM. No binding was observed to any other monosaccharide with the same linker, including GlcNAc-β, Gal-β, and Glc-α. In addition, no binding was observed to any other glucans, including ones composed of Glcβ1–2Glc, Glcβ1–3Glc, or Glcβ1–4Glc repeats, but some moderate binding was observed to Glcβ1–4Man at higher concentrations. Thus, the antibody displays very high selectivity for a narrow set of glycans. A possible biological target could be β1–6 linked glucans, which were not on our array.

### Antibodies targeting glycans present in both mammals and microbes

Bacteria and other microbes often produce glycans that are identical or nearly identical to mammalian glycans. Some examples include Lewis antigens, blood group antigens, and various globosides. We isolated several antibodies that recognized these types of glycans.

#### ADI-47319, ADI-47217, and ADI-45440, hyaluronic acid–binding antibodies

Hyaluronic acid is an anionic glycosaminoglycan polymer composed of alternating GlcNAcβ1–4GlcAβ1–3 residues. It is found in the extracellular matrix of a variety of human tissues, such as connective tissues. In addition, it is present on a variety of bacteria, including *Pasteurella multocida*, *Cryptococcus neoformans*, *Streptococcus pyogenes*, *Streptococcus uberis*, *Escherichia coli*, and *S. pneumoniae A and C* (48).

ADI-47319 and ADI-47217 had similar selectivities (Figs. 4 and S4). Antibody ADI-47319 was highly selective for hyaluronic acid oligosaccharides with a terminal glucuronic acid, such as a hyaluronic acid nonasaccharide Hya9 (GlcAβ1–3GlcNAcβ1–4GlcAβ1–3GlcNAcβ1–4GlcAβ1–3GlcNAcβ1–4GlcAβ1–3GlcNAcβ1–4GlcAβ). It did not bind at all to hyaluronic acid oligosaccharides of similar length but containing a terminal GlcNAc at the nonreducing end instead of a GlcA. While a terminal GlcA was necessary, it was not sufficient. For example, ADI-47319 did not bind GlcAβ1–3Galβ1–3GlcNAcβ1–3Galβ or heparosan oligosaccharides such as GlcAβ1–4GlcNAcα1–4GlcAβ1–4GlcNAcα1–4GlcAβ1–4GlcNAcα1–4GlcAβ1–4GlcNAcα1–4GlcAβ, demonstrating a requirement for the β1–3 linkage and a GlcNAc as the second residue. ADI-47319 bound to Hya7 and Hya9 with an estimate apparent *K*_*D*_ value of 20 nM. We are not aware of any mAbs with similar selectivity as ADI-47319 or ADI-47217.

In contrast to the aforementioned antibodies (ADI-47319 and ADI-47217), antibody ADI-45440 bound best to hyaluronic acid oligosaccharides with a terminal GlcNAcβ, such as hyaluronic acid octasaccharide Hya8 (GlcNAcβ1–4GlcAβ1–3GlcNAcβ1–4GlcAβ1–3GlcNAcβ1–4GlcAβ1–3GlcNAcβ1–4GlcAβ). Antibody ADI-45440 bound to Hya6 and Hya8 very tightly on our array, with an estimate apparent *K*_*D*_ value of <5 nM for both oligosaccharides. No binding was observed to hyaluronic acid oligosaccharides with a terminal GlcA residue, rather than GlcNAc, indicating that the terminal residue is a critical recognition element. In addition to differences in terminal monosaccharide preference, ADI-45440 also displayed much broader reactivity than ADI-47319. ADI-45440 bound to chitin oligosaccharides (GlcNAcβ1–4GlcNAcβ oligomers), cellulose oligomers (Glcβ1–4Glcβ1), and Fuc-α monosaccharide. Based on its binding to chitin oligosaccharides on the array, we interrogated ADI-45440 against chitin-coated plates and found it to bind well. We are not aware of any mAbs with similar selectivity as ADI-45440.

Antibodies that recognize hyaluronic acid could potentially cause autoimmune reactions; however, these antibodies were isolated from healthy subjects. Our working hypothesis to explain this discrepancy is that these antibodies can bind hyaluronic acid when presented on the surface of a bacterial cell but not human hyaluronic acid that would be present in the extracellular matrix; thus, they would not be considered autoantibodies. Since the antibodies are specific for the nonreducing terminus and likely require a divalent complex to achieve tight binding, the presentation of the hyaluronic acid would be critical for recognition. Bacterial capsules present a high density of nonreducing termini all over the surface, whereas human hyaluronic acid in the extracellular matrix may only infrequently present two nonreducing termini in close enough proximity and with proper orientation to form a divalent complex with the two Fab arms of an antibody.

Our initial efforts to validate binding are consistent with this model. Antibodies ADI-47319 (terminal GlcA) and ADI-45440 (terminal GlcNAc) did not bind to plates coated with commercial hyaluronic acid samples. However, binding was observed to the bovine mastitis causative pathogen *S. uberis*, which produces a hyaluronic acid capsule. *S. uberis* Diernhofer (ATCC; catalog no.: 19436) was cultured in brain heart infusion broth (BHI), washed, fixed, and plated onto ELISA plates. The plates were incubated with ADI-47319, and negative control trastuzumab, and a positive control sheep antihyaluronic acid polyclonal antibody (Bio-Rad). As shown in Figure 6*C*, both ADI-47319 and the sheep antihyaluronic acid antibody produced significantly greater signals than the negative control. While additional studies will be needed to more fully validate this hypothesis, our results provide some initial evidence to support it.

#### ADI-47201, a heparosan-binding antibody

Heparosan is a nonsulfated glycosaminoglycan composed of repeating GlcNAcα1–4GlcAβ1–4 units. In eukaryotes, heparosan is the biosynthetic precursor for heparan sulfate, a key component of the extracellular matrix. Heparan sulfate is a heterogenous polymer that contains regions that are identical to heparosan; therefore, stretches of heparosan can be found in the extracellular matrix and other parts of the body (49). Heparosan is also present in the capsule of a variety of bacteria, including *E. coli* and *Pasteurella multicida* (50).

Antibody ADI-47201 bound heparosan oligosaccharides with a terminal GlcNAcα (Fig. 4), such as Hep-NAc-Tetra-06 (GlcNAcα1–4GlcAβ1–4GlcNAcα1–4GlcAβ) but not related heparosan oligosaccharides of similar length with a terminal GlcAβ such as Hep-NAc-Nona-04 (GlcAβ1–4GlcNAcα1–4GlcAβ1–4GlcNAcα1–4GlcAβ1–4GlcNAcα1–4GlcAβ1–4GlcNAαa1–4GlcAβ). Thus, the nonreducing end residue plays a critical role in recognition. However, a simple GlcNAcα monosaccharide was insufficient for binding, indicating that the second sugar (or more) is required for recognition. The antibodies also did not bind GlcNAcα1–4Gal, indicating that the identity of the second sugar is also important for binding. Antibody ADI-47201 had an estimated apparent *K*_*D*_ value of 20 nM. We are not aware of any mAbs that have similar selectivity as ADI-47201.

#### ADI-45474, a globoside-binding antibody

Globosides and isoglobosides are a family of glycolipids produced by mammals. Globosides contain the core structure Galα1–4Galβ1–4Glcβ-(Gb3), whereas isoglobosides contain the related sequence Galα1–3Galβ1–4Glcβ-(iso-Gb3). These trisaccharides can be modified with a GalNAc residue to produce Gb4 (GalNAcβ1–3Galα1–4Galβ1–4GlcNAcβ) and iso-Gb4 (GalNAcβ1–3Galα1–3Galβ1–4GlcNAcβ). Additional glycosylations lead to other family members, including Gb5/iso-Gb5, Globo H, the Forssman antigen, and stage-specific antigen 4. The globo series glycolipids are involved in a wide range of biological processes and are often overexpressed in cancers (51, 52, 53). In addition, various microbes produce the glycan portion of globosides/isoglobosides.

Antibody ADI-45474 selectively bound Gb4 and iso-Gb4 (Fig. S4). The best glycan was Gb4, with an estimated apparent *K*_*D*_ value of 40 nM. Some minimal binding was observed to GalNAcβ monosaccharide and a glycopeptide containing a GalNAcβ residue linked to the side chain of tyrosine. However, no binding was observed to the precursor trisaccharides, Gb3 and iso-Gb3, or to the other globo series family members listed previously. In addition, no binding was observed to the GalNAcβ terminal glycolipid asialo-GM2 (GalNAcβ1–4Galβ1–4Glc), the related GalNAcβ terminal gangliosides (GM2, GD2, GT2, and GQ2), or LacDiNAc (GalNAcβ1–4GlcNAc). To further validate recognition of Gb4, Gb4-ceramide was adsorbed onto hydrophobic ELISA plates and a serial dilution of ADI-45474 was evaluated in an ELISA format. The antibody ADI-45474 bound to the Gb4-ceramide–coated plates as shown in Figure 6*D*.

#### ADI-47119, ADI-47180, and ADI-47213, antibodies that target ABO blood group and Lewis antigens

The ABH blood group antigens are a set of carbohydrates that define the major blood types. These glycans, as well as the related Lewis antigens, are expressed on red blood cells and a variety of other tissues within the body (54, 55). Blood group and Lewis antigens are also expressed on a variety of bacteria and viruses. Individuals develop antibodies to blood group antigens that are not present in their body, as those glycans are viewed by the immune system as foreign.

ADI-47119 bound the Lewis C antigen and several closely related glycans that contain a terminal Galβ1–3GlcNAcβ disaccharide motif (Fig. S5), including lacto-*N*-tetraose, linear lacto-*N*-hexaose, and branched lacto-*N*-hexaose. The disaccharide displayed equivalent binding as larger glycans, indicating that a disaccharide is sufficient for recognition. Antibody ADI-47119 had an estimated apparent *K*_*D*_ value of 50 nM for the disaccharide. Glycans with a terminal Galβ1–3GlcNAcβ disaccharide are overexpressed in a variety of cancers, and mAbs that target this glycan display promising anticancer activity in preclinical studies (56, 57).

ADI-47180 bound Lewis B (Fucα1–2Galβ1–4(Fucα1–3)GlcNAc) with an estimated apparent *K*_*D*_ value of 100 nM (Fig. S4). Some minimal reactivity with Lewis Y was also observed, but not binding to Lewis A (a substructure of Lewis B) was observed. This antibody also bound tightly to several synthetic derivatives of Lewis Y, with an estimated apparent *K*_*D*_ value of 10 nM. Lewis B is expressed on *Helicobacter pylori* and has altered expression on cancer cells (58, 59).

Antibody ADI-47213 displayed high selectivity for the blood group B antigen, a trisaccharide with the sequence: Galα1–3(Fucα1–2)GlcNAc (Fig. S4). No binding to the A antigen was observed, even though it is very similar in sequence: GalNAcα1–3(Fucα1–2)GlcNAc. In addition, no binding was observed to the blood group H antigen (Fucα1–2GlcNAc), a substructure of the B antigen. The antibody bound blood group B with an estimated apparent *K*_*D*_ value of 150 nM. This antibody was derived from an IgM memory B cell and would likely bind tighter as an IgM in serum because of avidity effects.

### Antibodies targeting plant glycans

#### ADI-47173, a xylan/cellulose-binding antibody

Both xylans and celluloses are major structural components of plants. In addition, cellulose is an exopolysaccharide found in bacterial biofilms (60). These glycans are composed of Glcβ1–4Glcβ (cellulose) or Xylβ1–4Xylβ (xylan) repeating units. From a structural point of view, both d-xylopyranose and d-glucopyranose have the same stereochemical arrangement of hydroxyls (all equatorial), with the difference being the presence or the absence of the C6 group. Antibody ADI-47173 displayed high selectivity for glycans containing either Glcβ1–4Glcβ or Xylβ1–4Xylβ motifs (Fig. S4). The best ligands had at least three xylose or glucose residues. ADI-47173 bound these glycans on the array with an apparent *K*_*D*_ value of 40 nM.

The antibody ADI-47173 was evaluated against a range of natural glycans including xylan (Beechwood, Xylβ1–4Xyl backbone with ∼13% α1–2GlcAOMe branches), lichenan (Icelandic moss, Glcβ1–4Glcβ1–3Glcβ1–4), and xyloglucan (tamarind, Glcβ1–4Glc backbone with α1–6 Xyl branching). An initial screen revealed binding to both xylan and lichenan. Serial dilutions of the antibody evaluated by the glycan ELISA format revealed strong interactions between ADI-47173 and both xylan and lichenan (Fig. 6*E*).

#### ADI-47073, a galactan-binding antibody

Galactans are polysaccharides composed of linear galactose polymers connected *via* β1–3, β1–4, or β1–6 glycosidic linkages. Galactans are key components of plant cell walls, and changes to these glycans occur during various biological processes affecting cell wall properties, such as fruit ripening (61). Antibody ADI-47073 bound best to Galβ1–4Galβ but also bound Galβ1–3Galβ and to a lesser extent Galβ1–6Galβ (Fig. 5). The estimated apparent *K*_*D*_ value for Galβ1–4Galβ was 40 nM. No binding was observed to any other glycan on the array, including Galβ1–4Glc, Galβ1–4GlcNAc, or Galβ1–6Man. We also tested antibody ADI-47073 for binding to commercially acquired galactan. As shown in Figure 6*F*, ADI-47073 bound to the galactan-coated plates significantly higher than the trastuzumab control.

#### ADI-45393, an antibody that binds linear beta mannan

Beta-mannans are major components of plant cell walls, especially softwoods and seeds of many plants, as well as some algae (62). Antibody ADI-45393 bound to linear beta 1–4 linked mannose oligomers (Fig. 5) but not galactomannan (linear mannan with galactose branches) or glucomannan oligosaccharides (mixture of beta 1–4 linked glucose and mannose). The affinity of this antibody was modest, with an estimated apparent *K*_*D*_ value of 150 nM. This antibody was derived from an IgM memory B cell, and binding of an IgM version of this antibody would likely be much tighter because of higher avidity. We note that the array only contains beta 1–4 linked mannose; other linkages such as beta 1–2 mannans were not evaluated.

### Antiglycan antibodies selected in our screen had low polyreactivity scores and normal hydrophobicity

Most of the antibodies used in our screen had been evaluated in a polyreactivity assay that measures binding to a mixture of soluble membrane protein and soluble cytosolic protein fractions from Chinese hamster ovary cells (31, 63). Based on the polyselectivity reagent (PSR) scores, antibodies were predicted to have no (<0.1), low (between 0.1 and 0.33), or high (>0.33) polyreactivity. Approximately 14% of the 516 antibody panels had a polyreactivity score of ≥0.1 (58 with low PSR scores; 11 with high PSR scores). Thus, a random selection of 26 antibodies from our panel would be expected to include about four antibodies with a polyreactive score ≥0.1. In our case, five of the initial 26 hits had a polyreactivity score of ≥0.1 (Table 1). Of those five, two antibodies were weak binders, two were polyreactive in our assay, and one was an inconsistent positive in our screen. None of the 17 antibodies with apparent *K*_*D*_ values ≤150 nM had a score over 0.1. Thus, most of the antibodies we identified do not bind widely to the diverse assortment of antigens present in the polyreactivity assay. HIC retention times were also available for the antibodies (Table 1). HIC retention times are often used to assess the hydrophobicity of an antibody and evaluate potential risks for precipitation/aggregation and nonspecific interactions. None of the 17 hits had a delayed retention time, but one of the weak binding antibodies and one of the polyreactive antibodies identified in our screen did. There was no statistically significant difference between the melting temperatures of antiglycan antibodies and the other antibodies from the panel. In addition to the polyreactivity assay, our array also contains DNP-BSA and dsDNA, two molecules that are often bound by polyreactive antibodies. None of the 22 glycan-specific antibodies bound to either of these two components, further confirming that they are not polyreactive. The two polyreactive antibodies from the 26 original hits bound DNP but not dsDNA.

Taken together, the polyreactivity data, HIC retention times, and glycan microarray data demonstrate that most of the antiglycan antibodies identified in the screen have good to excellent selectivity. In addition, the data suggest that either most polyreactive antibodies do not bind well to glycans on our array or our screening protocol (*i.e.*, elimination of antibodies that bind to highly charged glycosaminoglycans, Galα1–4Gal-CETE, and/or KDOα2-8KDOα2-4KDOα) filters them out. In either case, the results verify that the binding events observed in our screen are not because of polyreactivity.

### Antiglycan antibodies have similar germline gene usage as the overall panel but shorter complementary determining region 3 of the heavy chain lengths

In addition to analysis of polyreactivity, we also evaluated gene usage, mutation rates, and complementary determining region 3 of the heavy chain (CDRH3) lengths for the 17 best hits *versus* the overall panel of antibodies (Table 2). Overall, the antiglycan antibodies were very similar to the full panel of antibodies. For example, VH gene usage, kappa and lambda proportions, and somatic hypermutation rates for both the heavy chain (HC) and light chain (LC) did not display statistically significant differences for hits *versus* the full panel. The only significant difference was the length of CDRH3. The glycan-binding antibodies had significantly shorter CDRH3s (median of 12 residues for the hits *versus* 15 residues for the panel; *p* = 0.001). This feature is consistent with previous observations that antiglycan antibodies with charged epitopes have shorter CDRH3s when compared broadly with human mAbs (64). Nevertheless, the group of hits is relatively small, and additional studies will be needed to further evaluate this correlation.

**Table 2:** Sequence analysis of human monoclonal panel

Category	Glycan-binding antibodies	Nonglycan-binding antibodies	*t* test	ρ
CDRH3 AA length (mean)	13.41	15.82	2.7399	0.0064
CDRL3 AA length (mean)	9.45	9.60	0.5866	0.5577
Lambda	31.8%	31.3%	ND	ND
Kappa	68.2%	68.8%	ND	ND
VH NT mutations (mean)	13.14	9.91	1.764	0.0784
VL NT mutations (mean)	8.27	6.82	1.061	0.2891
VH AA mutations (mean)	10.23	8.23	1.411	0.1588
VL AA mutations (mean)	4.96	4.51	0.4908	0.6238

Abbreviation: ND, not determined.

### Antiglycan antibodies were enriched in the IgG memory B-cell compartment

One of our objectives of this study was to gain insight into whether antiglycan antibodies are enriched in certain B-cell subsets. Therefore, we analyzed the surface markers expressed by the B cells from which the 17 antiglycan antibodies were identified (Table 3). The majority of these antibodies were isolated from IgG memory B cells. IgG memory B cells accounted for 32% of the starting panel and 65% (11/17; *p* = 0.01) of the hits. Long-lived plasma cells accounted for the second highest source of the hits, with 24% (4 of 17) of these antibodies displaying reactivity with glycan antigens. This percentage was slightly lower than the panel as a whole (34%). The other two hits were derived from IgM memory B cells, which represented 19% of the starting panel but only 11% of the hits. None of the glycan-binding antibodies were derived from naïve B cells, early emigrant B cells, or immature B cells, which comprised about 15% of the starting panel. Taken together, the results indicate that the human IgG memory B compartment in our panel is preferentially enriched in antiglycan antibodies relative to other human B-cell subsets considered in this study.

**Table 3:** B-cell subset analysis of human monoclonal panel

IgG source	Pop.	Pop. %	Hits (%)	Hit (%)/Pop (%)	Good hits (%)	Good hit (%)/Pop (%)
IgG memory	169	32.8	12 (50%)	1.52	11 (65%)	1.98
LLPCS	175	33.9	7 (29%)	0.86	4 (24%)	0.71
IgM memory	95	18.4	5 (21%)	1.1	2 (12%)	0.65
Naïve	41	8.0	0	0	0	0
Early emigrant cells from bone marrow	24	4.7	0	0	0	0
Early immature cells	12	2.3	0	0	0	0
Total	516		24 (5.4%)			

Abbreviation: Pop., population.

## Discussion

Antiglycan antibodies are an important but understudied class of antibodies. Endogenous antiglycan antibodies play key roles in host defense, and new information about their origins, development, and properties would enhance our fundamental understanding of the immune system. In addition, unique antiglycan antibodies can arise during infections, autoimmune diseases, and vaccination. Therefore, the presence or the absence of certain antiglycan antibody populations can provide valuable information about a patient’s health status, making them appealing targets for diagnostic applications. Information about their properties can help in the design and development of detection strategies. In addition to endogenous antibodies, monoclonal antiglycan antibodies are extremely useful as therapeutic agents, diagnostics, and basic research tools. While critically important, we know very little about carbohydrate-binding antibodies, primarily because of a lack of access to individual well-characterized antibodies.

To address these challenges, we developed a glycan microarray screening approach to identify antiglycan antibodies to a wide variety of carbohydrate antigens. Using the screen, we evaluated binding of over 500 antibodies to approximately 800 antigens, encompassing about 400,000 potential antibody–antigen interactions. Using this approach, we identified a variety of new human antibodies to a diverse assortment of carbohydrate antigens.

Our results provide new insights into antiglycan antibody–binding properties, biophysical characteristics, and sequences. Antiglycan antibodies are often considered to have weak affinity and poor specificity. While some of the antibodies we identified were consistent with that view, others displayed high-affinity binding with apparent *K*_*D*_ values in the low nanomolar range. In addition, the majority of antiglycan antibodies identified in our screen exhibited good to excellent selectivity. For example, antibody ADI-47319 bound exclusively to a subset of hyaluronic acid oligosaccharides with a GlcAβ1–3GlcNAc at the nonreducing end. In contrast, ADI-45440 bound hyaluronic acid but only when the glycan had a terminal GlcNAcβ1–4GlcA. These antibodies illustrate selectivity not only for a particular glycan but also for distinct epitopes within the glycan. In terms of biophysical properties, the glycan-binding antibodies had low polyreactivity, normal hydrophobicity, and normal stability relative to the full panel. Their sequence usage and mutation rates were also very similar to the overall panel.

Our results also provide insights into the frequency and origins of antiglycan antibodies. Overall, about 5% of the antibodies in the screened collection bound to a carbohydrate on our microarray, providing an estimate of the frequency of antiglycan antibodies in the B-cell subsets we studied. Since there are numerous carbohydrates in nature that were not present on our array, this frequency represents a lower limit and may be considerably higher. In addition to the overall frequency, our results also provide information about the relative frequencies in different B-cell subsets we tested. In our study, the majority of antiglycan antibodies were derived from IgG memory B cells, whereas none originated from naïve, early emigrant, or immature B cells. In fact, the frequency of antiglycan antibodies from IgG memory B cells was approximately twice as high as what was expected from the overall panel composition, indicating that we could potentially double our hit rate in the future by focusing our screen on antibodies derived from IgG memory cells. Likewise, IgG memory cells would also be a good source of mRNA for constructing an antibody library for *in vitro* selections. If constructing a library, inclusion of both human kappa and lambda LCs would be beneficial, since roughly 40% of the antibodies we identified used a lambda LC.

In addition to the new information, we also identified several mAbs that could be useful for basic research or clinical applications. For example, ADI-45379 binds dPNAG and could be useful for studying biofilm and/or inhibiting biofilm formation. More extensive studies on this antibody are ongoing. Several antibodies, including ADI-47319, ADI-45440, ADI-47201, and ADI-47198, recognize glycans for which there are no known mAbs. Thus, these antibodies could be useful new tools for studying the associated glycans.

We note several caveats to our study. First, we excluded signals to several array components to avoid false-positive results in the screen. As a result, we may have overlooked some antiglycan antibodies in the panel. In addition, the microarray assay gives only modest signals with weak binders, so antibodies with apparent affinities in the micromolar range may not have been detected. Difficulties detecting weak binding may be especially important for antibodies that are natively produced as IgM but were screened as IgG in our assay, such as those from IgM memory B cells, from naïve, early emigrant, or immature B cells. The decrease in avidity from a decavalent IgM to a divalent IgG could result in significantly weaker binding on the array and may have reduced our ability to detect antiglycan antibodies from these B-cell subsets. Since we did identify two antiglycan antibodies with apparent *K*_*D*_ values ≤150 nM from IgM memory B cells, the IgM to IgG switch does not preclude detection in our assay. These factors should be considered when interpreting our results. In addition, the set of newly discovered antibodies is relatively small. As a result, our understanding of binding properties, gene usage, mutation rates, and polyreactivity may evolve as we obtain more antibodies. Additional studies will be important to more fully evaluate our findings. Finally, certain types of B cells were not included in the study, such as marginal zone B cells and B1 cells. Marginal zone B cells are primarily located in the spleen, and B1 cells are primarily located in the peritoneal and pleural cavities. It is known that a portion of these B cells encode antiglycan antibodies (65, 66, 67). Therefore, the percentage of antiglycan B cells detected in our study is likely to be an underestimate. Also, the properties of antiglycan antibodies produced by marginal zone B cells and B1 cells may be different than the properties of antiglycan antibodies from LLPCs and memory B cells found in the bone marrow and blood. For example, B1 cells typically produce antibodies with little or no mutations; therefore, the affinities and selectivities of these antibodies are likely quite different from antibodies produced by LLPCs and memory B cells.

Finally, the results have several broader implications. Antiglycan antibodies are often thought to have weak affinity and poor selectivity. Most of the antibodies identified in our screen had good to excellent selectivity, demonstrating that the human immune system is capable of producing high-quality antiglycan antibodies. Therefore, the lack of good antiglycan antibodies may reflect a need for better methodology rather than a fundamental limitation of the immune system. Furthermore, these results and other prior results indicate that some, and possibly many, antiglycan antibodies present in human serum have good selectivity. In a few cases, we (68, 69) and others (70) have captured subsets of serum antiglycan antibodies using carbohydrate-based affinity resins and evaluated selectivity using glycan microarrays. In these cases, the polyclonal captured antibodies appeared to have high selectivity for the corresponding capture antigen. In addition, we have observed numerous cases where human serum contains IgG or IgM to a glycan but not a structurally related glycan. While instructive, analysis of polyclonal preparations is complicated. Results from this study further support this model by demonstrating high selectivity for an assortment of antibodies at the monoclonal level. The results also have implications for glycan microarray design. This study, and many prior studies, indicates that bacterial, fungal, and plant cell wall polysaccharides are a key focus of the humoral immune system. However, most glycan microarrays have fairly limited coverage of these types of carbohydrates. Expanding the number and diversity of microbial glycans could be beneficial, especially when profiling endogenous antibodies. In addition, several antibodies only bound when the glycans were presented at higher densities, highlighting the importance of presentation and indicating that variations in glycan density on the array surface are beneficial. With expanded diversity, multivalent scaffolds, and improved presentation, glycan microarrays will further enhance our understanding of antiglycan antibodies.

## Experimental procedures

### B-cell sorting and cloning of the antibodies

For full details on the preparation of the antibody panel, refer to the study by Shehata *et al.* (31). Blood samples and human bone marrow samples were obtained from healthy donors, as previously described (31). Isolated peripheral blood mononuclear cells were stained with CD19 (PECy7), CD3 (PerCP-Cy5.5), CD8 (PerCP-Cy5.5), CD14 (PerCP-Cy5.5), CD16 (PerCP-Cy5.5), CD27 (BV421), IgM (APC), CD10 (BV605), and IgG (BV605). Bone marrow was stained using antihuman CD19 (PECy7), CD3 (PerCP-Cy5.5), CD8 (PerCP-Cy5.5), CD14 (PerCP-Cy5.5), CD16 (PerCP-Cy5.5), CD138 (FITC), and CD38 (APC) (31). Single cells were sorted on a BD FACS Aria II (BD Biosciences) into plates containing lysis buffer. Human variable genes were amplified by reverse transcription and nested PCR using IgG- and IgM-specific cocktails (31). Amplified HC- and LC-paired variable regions were cloned into *S. cerevisiae via* homologous recombination and lithium acetate chemical transformation. Yeast colonies were picked and sequenced. In addition to 373 previously published antibodies, the panel consists of 143 new antibodies cloned from 23 early emigrant cells from bone marrow, 12 early immature cells, 33 IgG memory cells, 33 IgM memory cells, 38 LLPCs, and four naïve B cells (see Supporting Excel File). Human IgGs were expressed in *S. cerevisiae* for 6 days, and secreted IgGs were harvested and captured using protein A resin (MabSelect Sure from GE Healthcare Life Sciences). The resin was washed with PBS and eluted with 200 mM acetic acid (pH 3.5) and neutralized with 2 M Hepes (pH 8.0).

### Neoglycoprotein microarray fabrication

Microarrays were produced as previously described (29, 71). Three large arrays (eight duplicate arrays per slide) composed of 738, 816, and 873 neoglycoproteins, glycoproteins, glycopeptide conjugates, and controls were prepared and used for the experiments in this report. In addition, a smaller focused array (16 duplicates per slide) with 100 array components was fabricated. Array components were diluted to 125 to 200 μg/ml into print buffer (1× PBS buffer with 2.5% [v/v] glycerol, 0.0005% [v/v] Triton X-100, 0.0005 μg/ml soluble print dye [0.05 μg/ml Atto 532 (Sigma; catalog no.: 06699)] or 0.5 μg/ml Alexa Fluor carboxylic acid, Tris(triethylammonium) salt [Thermo Fisher Scientific; catalog no.: A33084]) and printed in duplicate onto SuperEpoxy 2 microarray substrate slides (ArrayIt). Spot morphology and the presence was confirmed by visualizing the soluble print dyes in a microarray fluorescence scanner (InnoScan 1100 AL; Innopsys). Binding profiles for a representative set of lectins and antibodies were used as quality control for each printed microarray batch. Printed microarrays were stored under vacuum at −20 °C. For full details on array fabrication, processing, and analysis, refer to protocol by Temme and Gildersleeve (72).

### Two-dimensional pooled strategy

Protein A purified mAbs were received in 96-well plates with a stock concentration median of 480 μg/ml. To conserve microarrays, a two-dimensional pooling strategy was employed. Two 8 × 12 plates with 192 samples were arranged and then pooled by rows (12 samples) and columns (16 samples). Two microliters of each sample was pooled into binding buffer (1× PBS [pH 7.4], 0.05% Tween-20 with 3% BSA) to a final volume of 105 μl. The final concentration of each sample in the pool varied between 2 and 30 μg/ml with a median value of 9.1 μg/ml. All two-dimensional pooled experiments were performed using the 738- or 816-component arrays.

### Microarray assay

The vacuum sealed bag with the microarray slides was removed from the −20 °C freezer and warmed to room temperature (RT) prior to opening. Slides were scanned in a microarray fluorescence scanner prior to blocking for quality control and grid alignment. An 8-well slides module (ProPlate Multi-Well Chambers; Grace Bio-Labs) was mounted onto each slide. Each well was blocked with 400 μl of blocking buffer (1× PBS [pH 7.4] with 3% BSA), and the slides were covered and stored overnight at 4 °C. The slides were brought to RT and washed 4 × 400 μl with wash buffer (1× PBS with 0.05% Tween-20, pH 7.4). The pooled samples were then added to the wells. For 192 mAbs in a 12 × 16 pooled strategy, 28 microarray wells (3.5 slides) were used. Slides were covered and incubated at 37 °C with gentle shaking for 3.5 h. Slides were washed with 3 × 400 μl wash buffer, then two additional 2-min wash cycles were employed. A cy3-labeled antihuman IgG secondary antibody (1:500 dilution in PBS with Tween-20 [PBST] + 3% BSA; for the 738 and 816-component arrays; Jackson ImmunoResearch, Cy3-conjugated AffiniPure Goat Antihuman IgG, Fcγ fragment specific with minimal crossreaction to bovine, horse, and mouse serum proteins, catalog number: 109-165-098, Lot no.: 148157; for the 873-component array: Jackson ImmunoResearch, Cy3-conjugated AffiniPure Goat Antihuman IgG, Fcγ fragment specific with minimal crossreaction to bovine, mouse, and rabbit serum proteins, catalog number: 109-165-170, Lot no.: 149053) was then applied to each well. Slides were covered and incubated at 37 °C with gentle shaking for 1.5 h. Slides were washed as before, then removed from the slide module, and submerged in wash buffer for 5 min. Slides were placed in a 50 ml conical tube and dried by centrifugation (swing bucket rotor, 5 min at 1500*g*). The slides were scanned with an InnoScan microarray fluorescence scanner at 5 μm resolution using the 532 nm laser for Cy3. To capture all possible antiglycan mAbs, photomultiplier tube gain settings of 50 and 10 were used for the pooled strategy. For individually run mAbs, photomultiplier tube gain settings of 10 and 1 were used. Full microarray data can be found in the Supporting Excel File.

### Microarray analysis and antiglycan mAb identification

Fluorescence intensity quantification was performed using GenePix Pro software (Molecular Devices) (72). Preassay images of the arrays were used to flag and remove any missing or corrupted spots and to align the grid for postassay feature quantification. Median background corrected relative fluorescence unit values were reported as the average of replicate spots. Signals greater than 10× background were marked as positive values. Positive values from combined rows that could be matched with a paired column were flagged as possible hits. All possible hits were then validated by running the mAb from the source plate individually on the array. See Supporting Excel File #1 for validation profiles on the 738 array (816 for mAb ADI-45379 and ADI-47299). Full microarray data can be found in the Supporting Excel File. Apparent *K*_*D*_ values were estimated from the four-concentration series following the method of Gordus and MacBeath (73).

### Sequence analysis

The sequences of the newly identified antiglycan antibodies can be found in the Supporting Excel File. Potential differences between antiglycan antibodies and the full panel (*e.g.*, mutation rates, kappa *versus* lambda usage, CDRH3 length) were evaluated using a *t* test or *z* test for two population proportions.

### Recombinant expression and purification of mAbs

To obtain sufficient stocks of selected antibodies and further confirm binding, transient transfection in Expi293 cells was utilized. Plasmid inserts were synthesized and cloned into expression plasmids by Genscript. Signal sequence for human IgG, MGWSCIILFLVATATGVHS (ATGGGCTGGAGCTGCATCATTCTGTTTCTGGTGGCCACAGCCACCGGCGTGCACAGC), was used for all plasmids. Plasmids for HC (pFuse-CHIg-hG1) and kappa and lambda LCs, pFuse-CLIg-hk, and pFuse-CLIg-hL2 were obtained from InvivoGen. ElectroMAX DH10B cells (Thermo) were transformed by electroporation and grown on selective antibiotic resistance plates. Single colonies were picked and expanded sequentially to 100 ml cultures (TB). About 100 ml overnight cultures were pelleted by centrifugation (15 min @3200*g*), and the plasmid DNA was extracted and purified by maxiprep (Zymo Research).

Expi293 cells were cultured in Expi293 serum-free media. For a 30 ml expression, 75 million cells were transfected using 80 μl expifectamine precomplexed with 15 μg of each HC and LC plasmids in 3 ml Opti-MEM serum-free media. Newly transfected cells were incubated in 125 ml flasks at 37 °C and 8% CO_2_ with 125 rpm shaking. Expi expression additives were added after 16 to 18 h according to the manufacturer’s recommended protocol. Cultures were harvested after 6 to 7 days. Cells and cellular debris were removed by centrifugation, and media were cleared by sequential filtration through 3.2, 1.6, and 0.45 μm syringe filters. The cleared media were diluted 1:1 with Protein A binding buffer (PBS, pH 7.0 + 0.02% NaN3 w/v). About 250 μl Protein A Resin (Genscript) was added to the buffered media. The mixture was incubated at RT with rotation for 3 h. Media/resin was loaded onto an Econopac column and washed 4 × 5 ml with Protein A binding buffer. Each mAb was eluted from the resin using 200 mM acetic acid (pH 3.5) and neutralized with 2 M Hepes (pH 8.0). mAb-positive fractions were concentrated in spin filters (Amicon Ultra; Millipore Sigma, 30 kDa MW cutoff) and buffer exchanged into mAb storage buffer (25 mM Hepes, 150 mM NaCl, pH 7.3). Antibody purity was determined by analytical HPLC SEC (Fig. S7). These antibodies were profiled on our 873-component microarray at four concentrations—see Supporting Excel File for full data.

### Protein aggregation studies

Recombinant antibodies isolated from supernatant by Protein A capture and acidic elution were further purified *via* FPLC system equipped with an SEC column (Cytiva; Superdex 200 Increase 10/300 GL) in PBS. IgG-positive fractions were combined and concentrated to ∼3 mg/ml. Trastuzumab-anns (Amgen; Lot no.: 1112132A) was also SEC purified prior to the experiment. Human IgG isotype control (InVivoMAb; catalog no.: BE0297; Lot no.: 786221N1) was used as is. A 100 μl aliquot of the SEC purified stock antibodies or isotype control were treated with 100 mM glycine (pH 2.5) for 5 min at RT. The antibodies were neutralized with 100 μl 1 M Tris (pH 8.0). The quenched antibodies were concentrated and buffer exchanged back into PBS using 30 K spin concentration columns (Amicon Ultra; Millipore Sigma, 30 kDa MW cutoff). The antibodies were diluted to 1 mg/ml based on nanodrop and analyzed for aggregation by analytical SEC (Fig. S1) and evaluated on the A873 array for off-target binding events (Fig. 2*B*).

Freeze–thaw cycling experiments were performed on antibodies at a constant protein concentration of 1 mg/ml. Three different buffer/additive combinations were prepared (PBS, PBS + 0.2 M trehalose, and PBS + 1% BSA). About 130 μl samples of each antibody in the different buffers were prepared. About 20 μl of each was preserved to serve as a negative control for the effects of freeze–thaw cycling. Each sample was frozen on dry ice for 5 min and then thawed in water for 5 min. After 1, 3, 5, 7, and 9 freeze–thaw cycles, 20 μl of each sample was taken. The antibodies were analyzed for aggregation by analytical SEC (Fig. S1) and evaluated on the A873 array for off-target binding events (Fig. 2*C*).

### Glycan ELISA

The following commercially sourced glycans were purchased: galactan (Megazyme; P-GALPOT), xyloglucan (Megazyme; P-XYGLN), xylan from beechwood (Megazyme; P-XYLNBE), lichenan (Megazyme; P-LICHN), chitin from shrimp shells (Sigma; C9752), chitosan oligosaccharide (Sigma; 523682), and Gb4-ceramide (Matreya LLC; Globosides). Except for Gb4-ceramide, which was dissolved to 10 μg/ml in methanol, all glycans were dissolved in Milli-Q water and diluted to a concentration of 100 μg/ml. About 50 μl of the diluted glycans were pipetted to each well of 96-well plates (Nunc; Multisorb). The glycans were dried in the plates by incubating the plates overnight at 37 °C. The glycan-coated plates were blocked with 200 μl of 3% BSA in PBS for 2 h at RT. ADI antibodies and control antibodies were serially diluted from concentrated stocks into 3% BSA in PBST to make 8-point dilution curves ranging from 200 to 0.2 μg/ml. The blocked plates were washed five times with PBST, and then 100 μl of the antibody samples were added to the plate in triplicate. After a 2 h incubation at 37 °C, the plates were again washed five times with PBST. To the washed wells was added 100 μl of a secondary antihuman IgG horseradish peroxidase (HRP) conjugate (Jackson ImmunoResearch Laboratories) diluted 1:5000 in 3% BSA in PBST. After a 1 h incubation at 37 °C, the plates were again washed five times with PBST. The plates were developed by incubating the wells for 5 min with 50 μl of 3,3′,5,5′-tetramethylbenzidine followed by quenching with 50 μl of 2 N H_2_SO_4_. A plate reader was used to measure the 450 nm abs of the plates. Resulting triplicate data were exported to excel and then analyzed in GraphPad Prism (GraphPad Software, Inc). See Supporting Excel File for full data.

### Cell ELISA

Bacterial strains *S. epidermidis* (Winslow and Winslow) Evans (ATCC; catalog no.: 35984) and *S. uberis* Diernhofer (ATCC; catalog no.: 19436) were cultured according to recommended procedures. *S. epidermidis* was cultured in tryptic soy broth or grown on tryptic soy broth–agar plates, whereas *S. uberis* was cultured in BHI or grown on BHI agar + 5% sheep’s blood. Single colonies were grown in 5 ml broth with shaking for 16 h. Overnight cultures were pelleted at 3200*g* for 10 min. The pellets were washed by resuspending in PBS and pelleting by centrifugation. Following three additional washes (2× PBS, 1× MilliQ), the pellets were resuspended in 50 ml cold methanol. To each well of a 96-well plate (NUNC; Maxisorb) was added 50 μl of the resuspended and fixed cells. The plates were spun to concentrate the cells in the bottom. Excess MeOH was removed by aspiration, and the plates were dried for 4 h at RT. The plated cells were rehydrated by adding Milli-Q water to the wells and incubating overnight at 4 °C. The plates were blocked with 200 μl of 3% BSA in PBS for 2 h at RT. The cell-coated plates were gently washed three times with PBS, followed by the addition of antibodies and controls serially diluted into 3% BSA in PBS. Primary antibodies were incubated on the plates for 2 h at RT with shaking. The plates were washed three times with PBS, followed by the addition of 100 μl of a secondary antihuman IgG HRP conjugate (Jackson ImmunoResearch Laboratories) or a secondary antisheep IgG HRP conjugate (Jackson ImmunoResearch Laboratories) diluted 1:5000 in 3% BSA in PBS. The plates were washed three times with PBS following a 1 h incubation with the secondary mAb at RT. The colorimetric assay was developed by incubating the wells for 5 min with 50 μl of 3,3′,5,5′-tetramethylbenzidine followed by quenching with 50 μl of 2N H_2_SO_4_. A plate reader was used to measure the 450 nm abs of the plates. Resulting triplicate data were exported to excel and then analyzed in GraphPad Prism. See Supporting Excel File for full data.

## Data availability

All data are available in the main article, supporting figures/tables, and supporting Excel file (full microarray data and antibody sequences).

## Supporting information

This article contains supporting information.

## Conflict of interest

The authors declare that they have no conflicts of interest with the contents of this article.

## Supporting information

Supporting Excel File
