# Supplementary material for: Microarray-guided evaluation of the frequency, B-cell origins, and selectivity of human glycan-binding antibodies reveals new insights and novel antibodies
Source: J Biol Chem. 2022 Sep 8;298(10):102468. doi: 10.1016/j.jbc.2022.102468 (PMC9576894; doi:10.1016/j.jbc.2022.102468)
Supplement: Supplemental Figures S1–S7 [file mmc2.pdf]

Supporting Information

for

Microarray-guided evaluation of the frequency, B cell origins, and selectivity of human ant-glycan antibodies reveals new insights and novel antibodies

J. Sebastian Temme[1], Jennifer A. Crainic[1], Laura M. Walker[2,3], Weizhun Yang[4a,b], Zibin Tan[4a,b], Xuefei Huang[4a,b,c], and Jeffrey C. Gildersleeve[1]*

[1]Chemical Biology Laboratory, Center for Cancer Research, National Cancer Institute, Frederick, MD, 21702. [2]Adimab LLC, Lebanon, NH 03766, USA. [3]Adagio Therapeutics, Inc., Waltham, MA 02451, USA. [4a]Department of Chemistry, Michigan State University, 578 S Shaw Lane, East Lansing, Michigan 48824, United States. [4b]Institute for Quantitative Health Science and Engineering, [4c]Department of Biomedical Engineering, Michigan State University, East Lansing, Michigan 48824, USA

*Corresponding author and Lead Contact: gildersj@mail.nih.gov

Short title: Analysis of human Anti-Glycan Antibodies

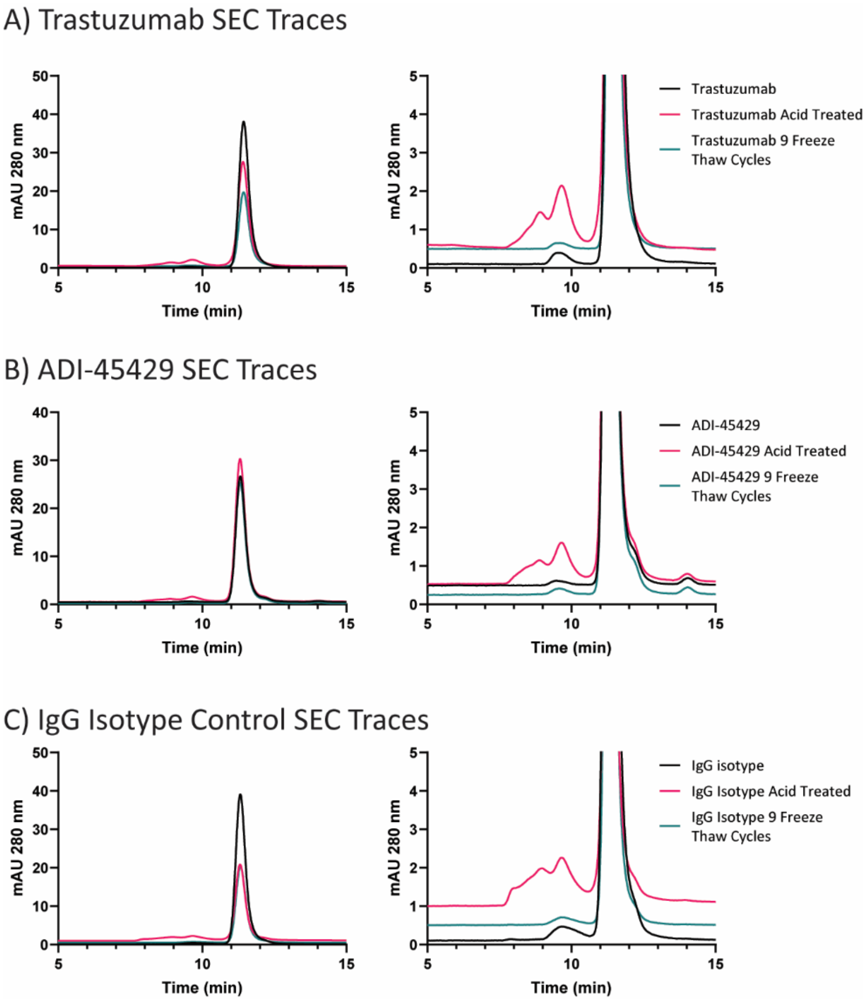

Figure S1. SEC traces of IgGs following acid treatment and freeze-thaw cycling. Characterization of the antibody aggregates by size-exclusion chromatography. Black trace – without treatment, red trace – 100 mM glycine pH 2.5 treatment, followed by quench, green trace – 9 freeze thaw cycles in PBS. A) Trastuzumab full spectrum on left, zoom over region of interest on left. B) ADI-45429 full spectrum on left, zoom over region of interest on left. C) IgG Isotype control from myeloma serum full spectrum on left, zoom over region of interest on left. TOSOH TSKgel SuperSW mAb HR 7.8 mm ID x 30 cm, 4 µm column with a mobile phase composed of 100 mM Na_2PO_4, 100 mM Na_2S0_4, 0.05% w/v NaN_3, pH 6.7. Flow rate of 0.75 mL/min. Signals in milli-absorbance units (mAU) measured at 280 nm.

A) Trastuzumab

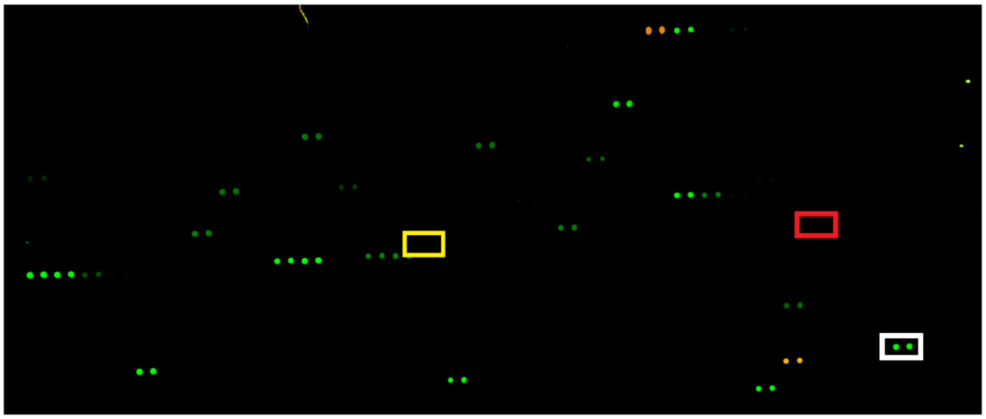

B) Trastuzumab after Freeze-Thaw Cycling

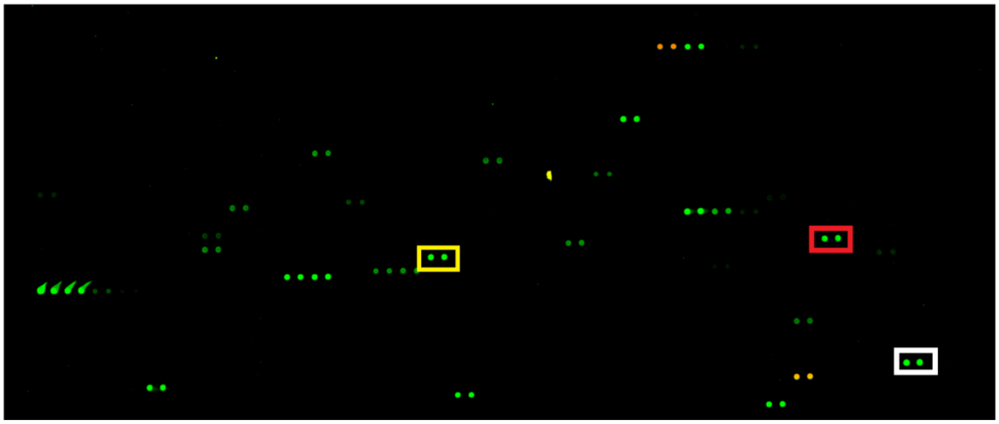

Figure S2. Trastuzumab on the 873 array. A)Trastuzumab, 50 µg/mL, Red Box – DNP-BSA (#481), Yellow Box – KDOa2-8KDOa2-4KDOa (#539), White Box – Her2 (#787). B)Trastuzumab, 9 Freeze-thaw cycles in PBS, 50 µg/mL, Red Box – DNP-BSA (#481), Yellow Box – KDOa2-8KDOa2-4KDOa (#539), White Box – Her2 (#787). Fluorescence images acquired on our 873-component glycan microarray.

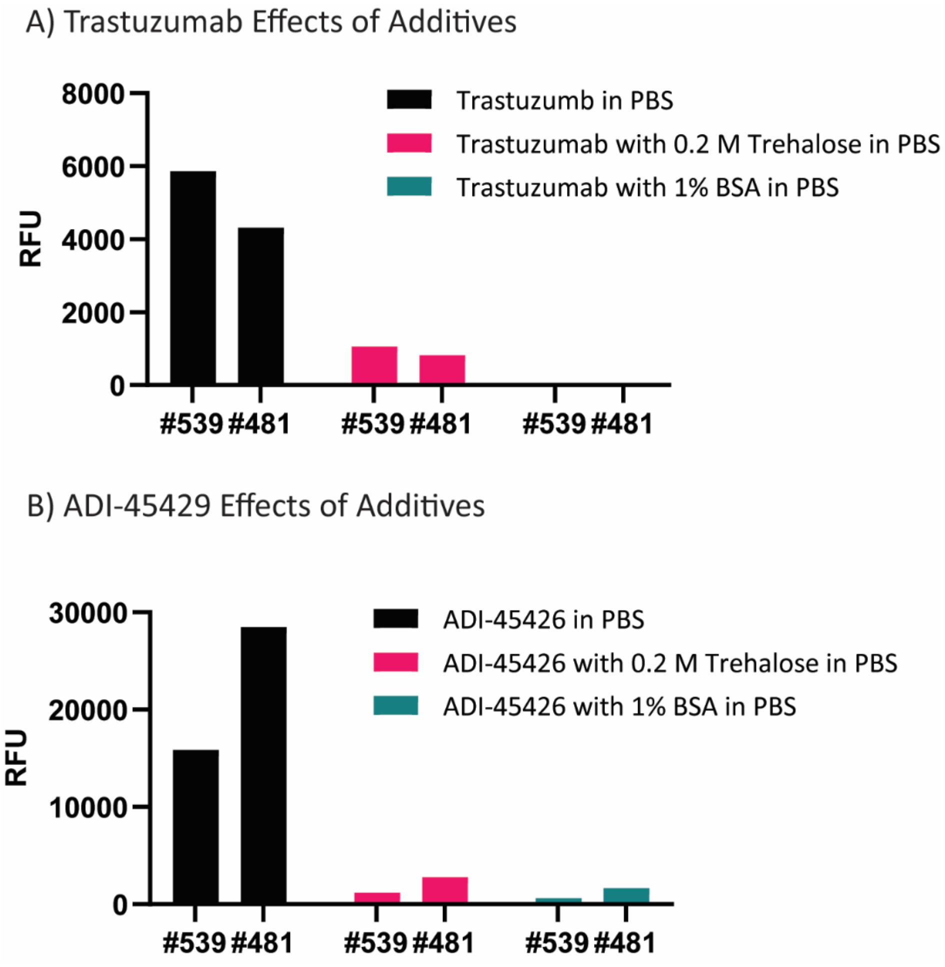

Figure S3. Signals for DNP-BSA (#481) and KDOa2-8KDOa2-4KDOa (#539) with Trastuzumab and ADI-45429 on the 873 array and the effects of stabilizing additives following freeze-thaw cycles. A) Trastuzumab, 50 µg/mL, 9 freeze-thaw cycles, PBS alone, PBS with 0.2 M trehalose, and PBS with 1% BSA. B) ADI-45426, 50 µg/mL, 9 freeze-thaw cycles, PBS alone, PBS with 0.2 M trehalose, and PBS with 1% BSA. Signals in relative fluorescence units (RFUs) for antibodies on our 873-component glycan microarray.

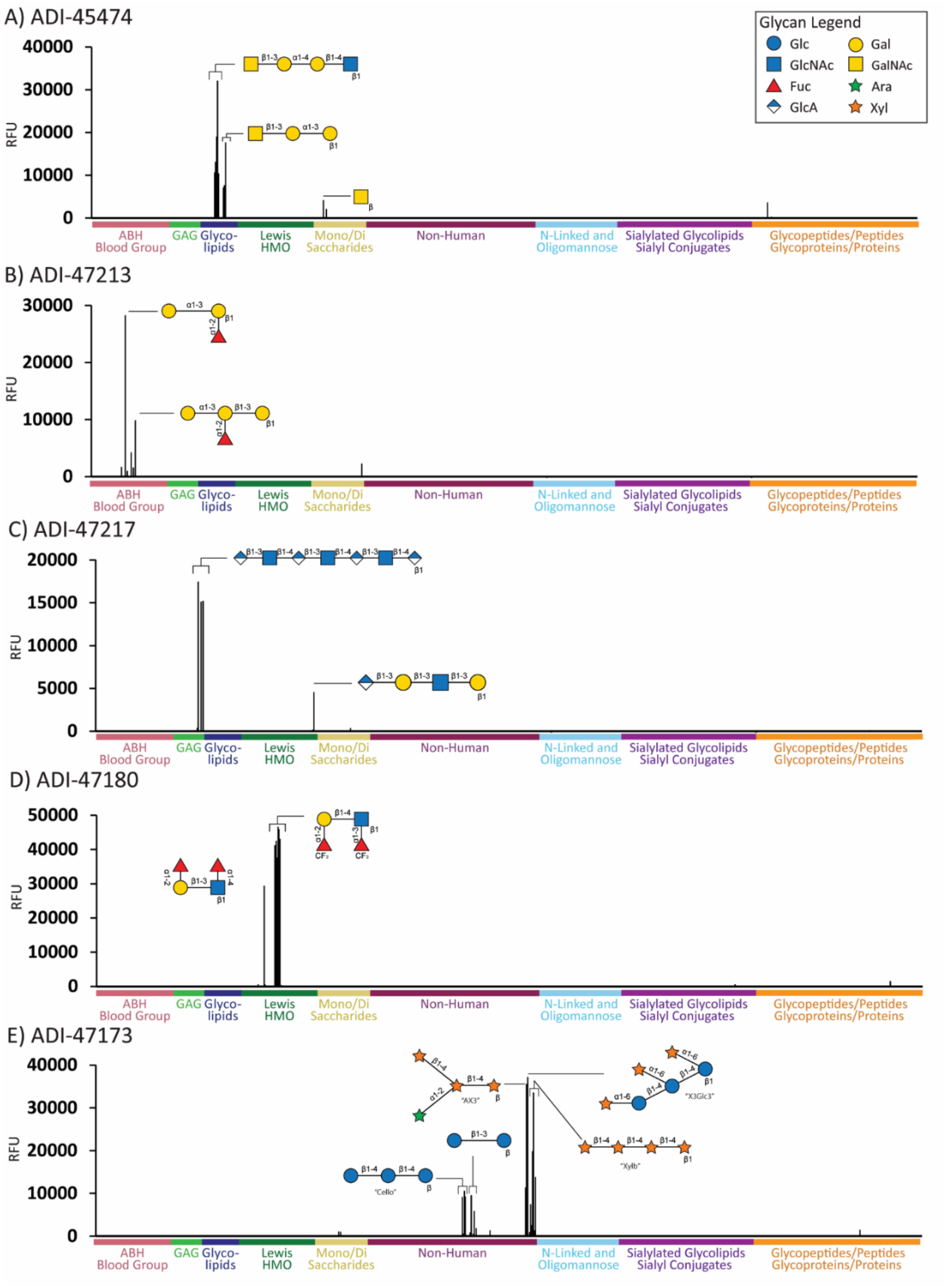

Figure S4. Binding profiles for ADI-45474, ADI-47213, ADI-47217, ADI-47180, and ADI-47173. Signals in relative fluorescence units (RFUs) for antibodies on our 873-component glycan microarray. Data shown at the following concentrations: ADI-45474 at 167 nM, ADI-47213 at 167 nM, ADI-47217 at 167 nM, ADI-47180 at 167 nM, and ADI-47173 at 167 nM. Positive and negative controls have been excluded. Glycan symbol structures were created in GlycoGlyph.

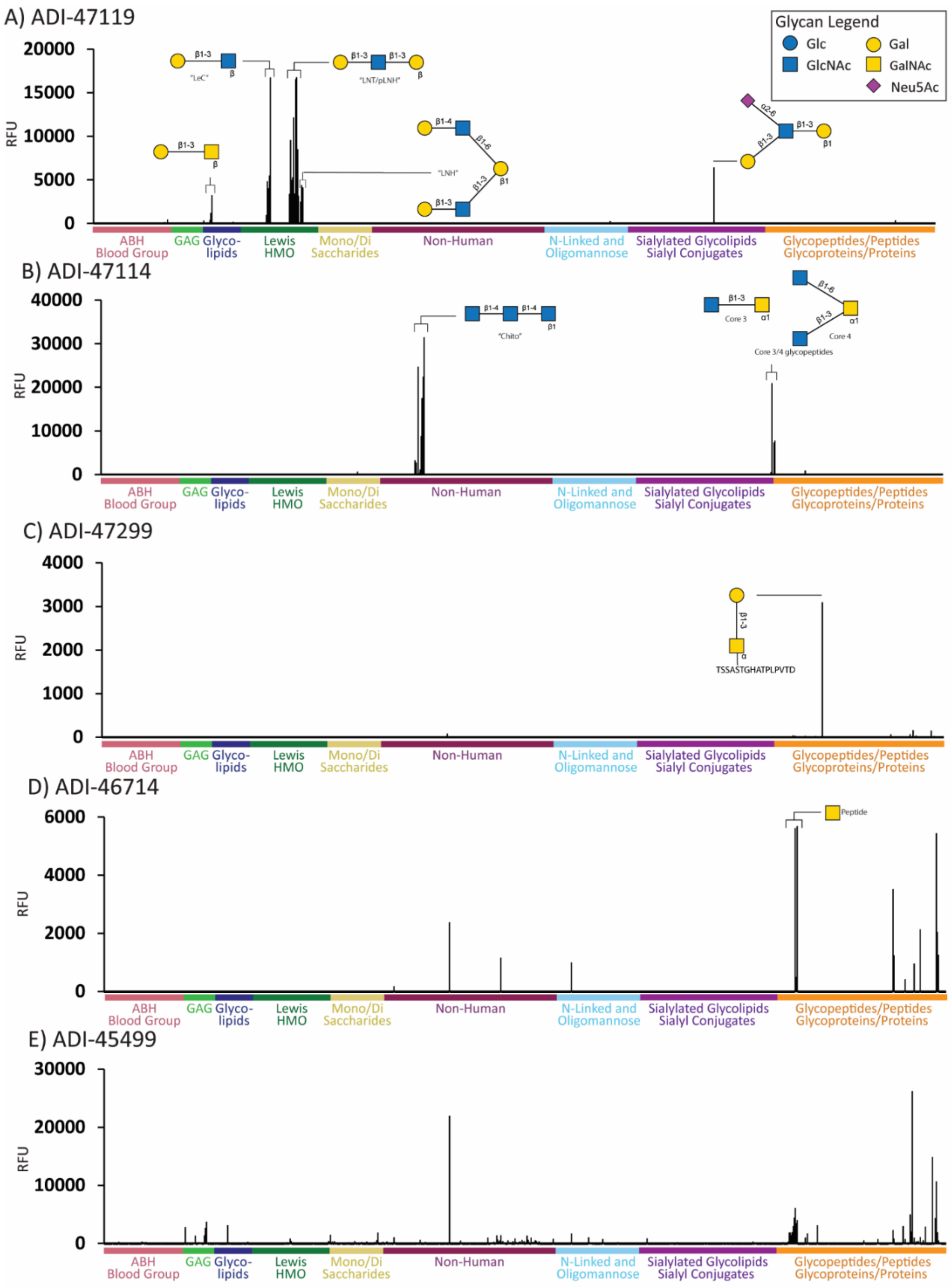

Figure S5. Binding profiles for ADI-47119, ADI-47114, ADI-47299, ADI-46714, and ADI-45499. Signals in relative fluorescence units (RFUs) for antibodies on our 873-component glycan microarray. Data shown at the following concentrations: ADI-47119 at 42 nM, ADI-47114 at 167 nM, ADI-47299 at 167 nM, ADI-46714 at 167 nM, and ADI-45499 at 167 nM. Positive and negative controls have been excluded. Glycan symbol structures were created in GlycoGlyph.

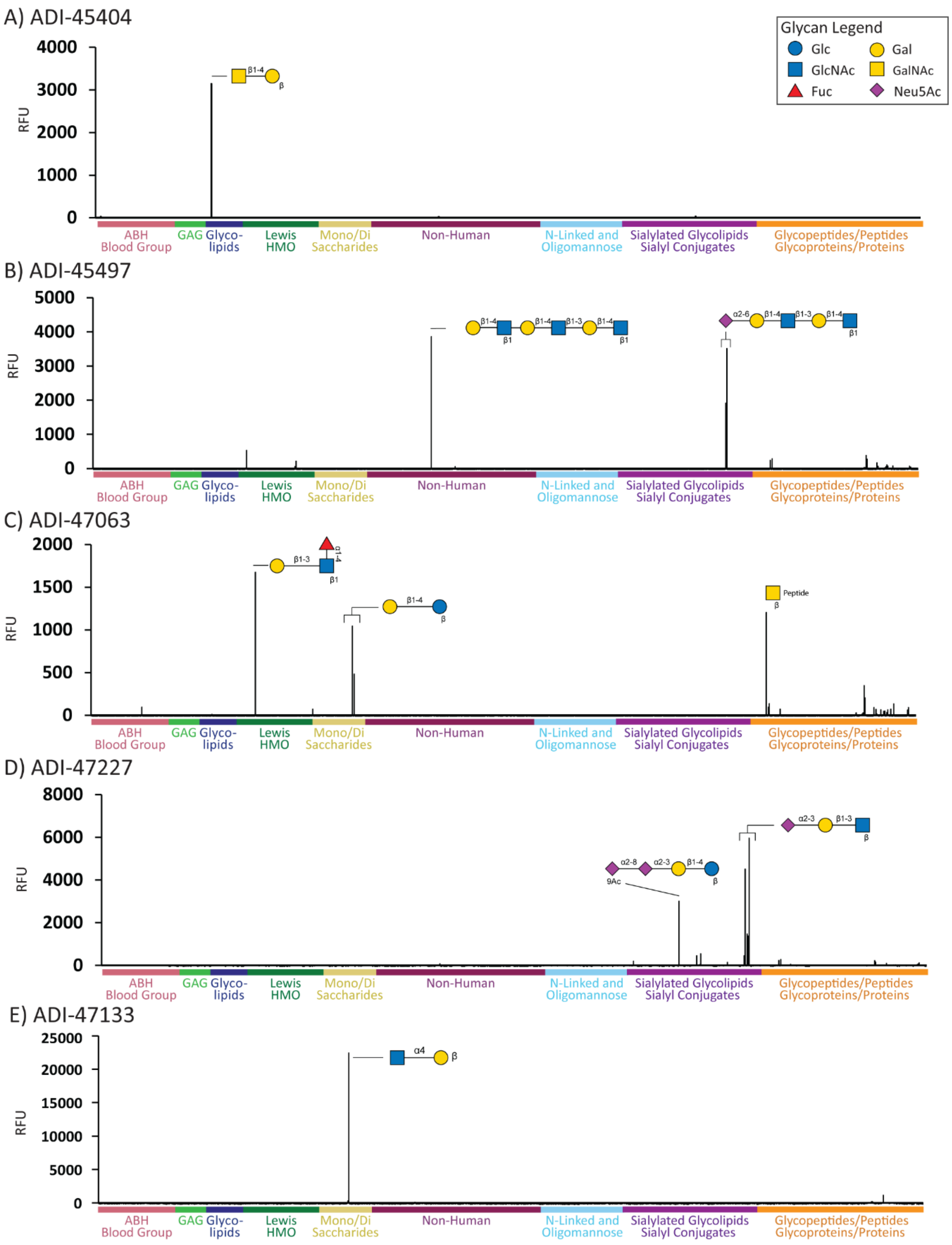

Figure S6. Binding profiles for ADI-45404, ADI-45497, ADI-47063, and ADI-47227. Signals in relative fluorescence units (RFUs) for antibodies on our 873-component glycan microarray. Data shown at the following concentrations: ADI-45404 at 667 nM, ADI-45497 at 167 nM, ADI-47063 at 167 nM, and ADI-47227 at 167 nM. Positive and negative controls have been excluded. Glycan symbol structures were created in GlycoGlyph.

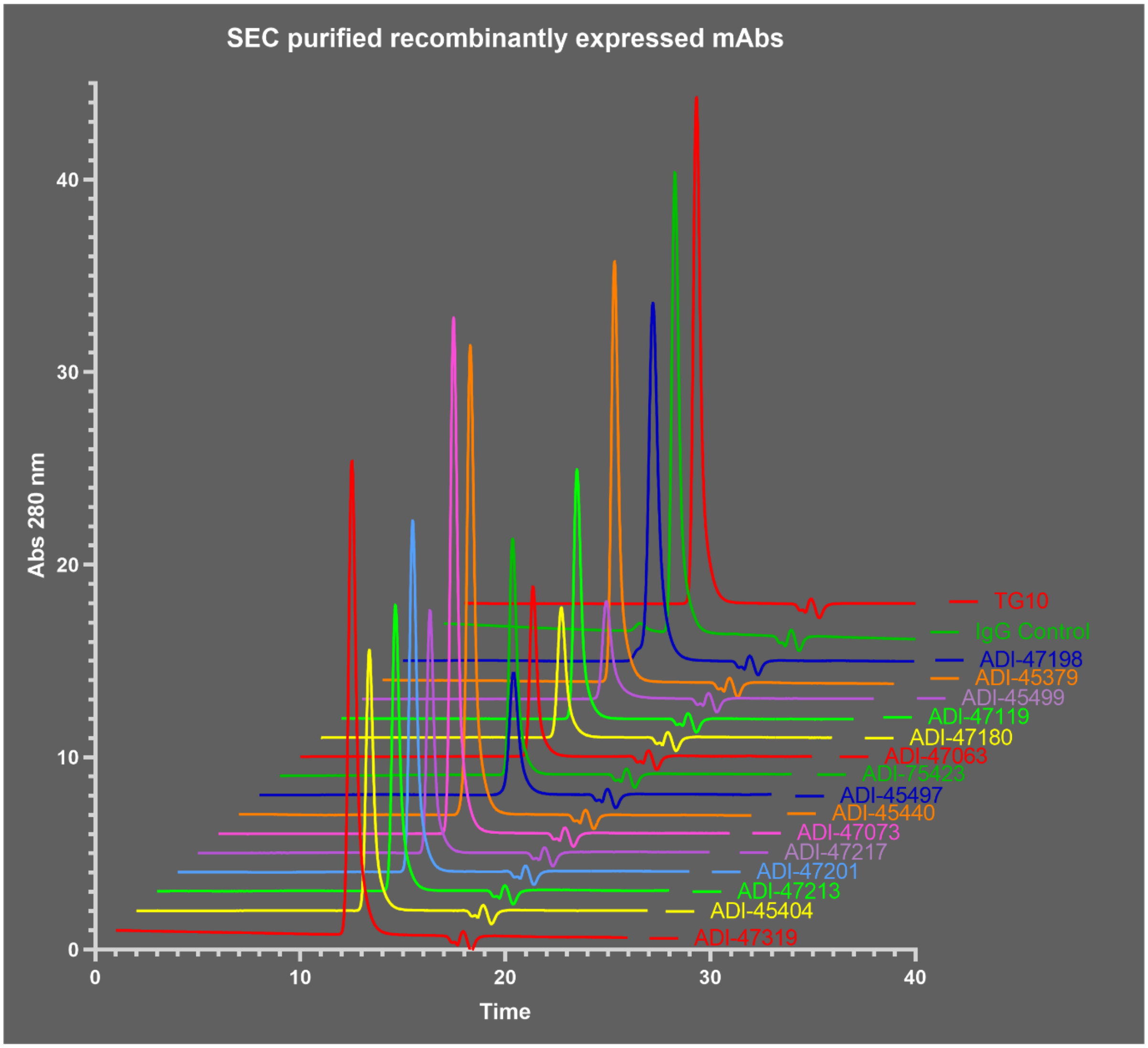

Figure S7. SEC traces of recombinantly expressed IgG mAbs identified in the HTS. Characterization of the recombinantly expressed antibodies by size-exclusion chromatography. TOSOH TSKgel G3000SWxl column with a mobile phase composed of 100 mM Na_2PO_4, 100 mM Na_2S0_4, 0.05% w/v NaN_3, pH 7.0. Flow rate of 0.75 mL/min.
